# Comparative toxicity assessment of in situ burn residues to initial and dispersed heavy fuel oil using zebrafish embryos as test organisms

**DOI:** 10.1007/s11356-020-11729-5

**Published:** 2020-12-03

**Authors:** Sarah Johann, Mira Goßen, Leonie Mueller, Valentina Selja, Kim Gustavson, Janne Fritt-Rasmussen, Susse Wegeberg, Tomasz Maciej Ciesielski, Bjørn Munro Jenssen, Henner Hollert, Thomas-Benjamin Seiler

**Affiliations:** 1 Department of Evolutionary Ecology and Environmental Toxicology, Goethe University Frankfurt, Max-von-Laue-Str. 13, 60438 Frankfurt am Main, Germany; 2 Department of Ecosystem Analysis, Institute for Environmental Research, RWTH Aachen University, Worringerweg 1, 52074 Aachen, Germany; 3 Department of Biology, Josip Juraj Strossmayer University of Osijek, Cara Hadrijana 8/A, 31000 Osijek, Croatia; 4 Department of Bioscience, Aarhus University, Frederiksborgvej 399, 4000 Roskilde, Denmark; 5 Department of Biology, Norwegian University of Science and Technology, 7491 Trondheim, Norway; 6 Ruhr District Institute of Hygiene, Rotthauser Straße 21, 45879 Gelsenkirchen, Germany

**Keywords:** AChE, Chemical dispersant, Embryo toxicity, EROD, In situ burning, Swimming behavior

## Abstract

In situ burning (ISB) is discussed to be one of the most suitable response strategies to combat oil spills in extreme conditions. After burning, a highly viscous and sticky residue is left and may over time pose a risk of exposing aquatic biota to toxic oil compounds. Scientific information about the impact of burn residues on the environment is scarce. In this context, a comprehensive ISB field experiment with approx. 1000L IFO 180 was conducted in a fjord in Greenland. The present study investigated the toxicity of collected ISB residues to early life stages of zebrafish (*Danio rerio*) as a model for potentially exposed pelagic organisms. The toxicity of ISB residues on zebrafish embryos was compared with the toxicity of the initial (unweathered) IFO 180 and chemically dispersed IFO 180. Morphological malformations, hatching success, swimming behavior, and biomarkers for exposure (CYP1A activity, AChE inhibition) were evaluated in order to cover the toxic response on different biological organization levels. Across all endpoints, ISB residues did not induce greater toxicity in zebrafish embryos compared with the initial oil. The application of a chemical dispersant increased the acute toxicity most likely due to a higher bioavailability of dissolved and particulate oil components. The results provide insight into the adverse effects of ISB residues on sensitive life stages of fish in comparison with chemical dispersant application.

**Supplementary Information:**

The online version contains supplementary material available at 10.1007/s11356-020-11729-5.

## Introduction

In case of an oil spill, the most effective response technologies have to be selected to minimize direct or indirect adverse effects on the environment. The selection of oil spill response measures, such as chemical dispersion, in situ burning (ISB), or oil recovery, strongly depends on operational parameters such as the physical-chemical properties of the spilled oil and the weather conditions on site (Ekperusi et al. 2019). Unique oil characteristics such as viscosity, density, pour point, and weathering status influence the oil fate and behavior as a function of temperature, wind conditions, and ice coverage (Spaulding 2017). Furthermore, the presence of sensitive organisms and the toxicity of treated as well as untreated oil are important information to evaluate the potential environmental impact by the oil itself and the side effects of applied oil spill response technologies (Wegeberg et al. 2017). Overall, a high efficiency is reported for chemical dispersants and ISB response technologies when applied under optimal conditions (Bejarano et al. 2014; Buist et al. 2013; Ekperusi et al. 2019; Lee et al. 2011).

In particular, ISB is discussed to be a suitable response technology to combat oil spills in Arctic seas (Buist et al. 2013; Faksness et al. 2012), as it requires little equipment and is applicable in ice-infested waters. By the ISB application, oil is removed from the water surface and converted into combustion products, which are emitted to the air, and burn residues left in the sea. The amount and nature of residues, brittle or highly viscous and sticky, depend on the oil type and burning efficiency (Fritt-Rasmussen et al. 2015). The nature of the ISB residues also influences whether they float on the water surface or sink. Even though the amount of residue is highly reduced compared with the initial volume of spilled oil, the residue may pose a risk for smothering seabirds or shorelines and might expose pelagic and benthic communities to toxic compounds (Fritt-Rasmussen et al. 2015) As reviewed by Fritt-Rasmussen et al. (2015) and Holmsen (2019), scientific information about the impact of ISB residues on the environment is scarce, and, in particular, research is needed on the aquatic toxicity of ISB residues.

Deviating from that, the application of a chemical dispersant, breaking down the oil slick into small droplets in the water column and preventing shorelines from smothering, is controversially discussed, since the acute toxicity toward aquatic biota in the water column is temporarily increased (Prince 2015).

To study the aquatic toxicity of different oil spill response measures, sensitive early life stages of fish can be used to assess potential effects of an oil spill occurring during spawning season. Impacts of petroleum products on fish embryos and larvae have been investigated extensively (de Soysa et al. 2012; Incardona et al. 2013; Jung et al. 2013; Nahrgang et al. 2016; Perrichon et al. 2016; Tairova et al. 2019). Even though sensitivities can vary among different freshwater and marine species (Incardona et al. 2014; Perrichon et al. 2016; Stieglitz et al. 2016), the observed phenotypic effects in the fish early developmental stages are markedly conserved (Incardona 2017; Incardona et al. 2014). Hence, the well-established ecotoxicological model species *Danio rerio* (zebrafish) was selected in the present study. The zebrafish is one of the best-studied fish species with many advantages including a fully sequenced genome (Howe et al. 2013), a well-described physiology and development, and, furthermore, it is easy to culture in the laboratory (Scholz et al. 2008; Strähle et al. 2012). Additionally, zebrafish early life stages have been extensively used to evaluate the toxicity of petroleum products (Incardona et al. 2013; Pauka et al. 2011; Perrichon et al. 2016; Philibert et al. 2016), and findings from those experiments were consistent with endemic species exposed to oil (Brette et al. 2014; Khursigara et al. 2017).

Within the framework of the EU-funded project GRACE (Jørgensen et al. 2019), a large-scale ISB field experiment with app. 1000 L heavy fuel oil (IFO 180) in a fjord close to Kangerluarsoruseq, Greenland, was performed (Wegeberg et al. 2018a). After the burning, the environmental effects were monitored focusing on tidal and pelagic communities. Additionally, ISB residues were collected from the water surface in order to characterize the ecotoxicological effects. In the present study, the toxicity of the ISB residues is assessed in laboratory experiments.

In order to assess acute embryo toxicity, in the present study, zebrafish early life stages were exposed to water-accommodated fractions (WAFs) from ISB residues. Additionally, embryos were exposed to WAFs prepared from the initial (untreated) IFO 180 and chemically dispersed IFO 180. By comparing the toxicity of those three WAFs, it was investigated whether ISB residue WAFs induced altered toxicity compared with WAFs of untreated and chemically dispersed heavy fuel oil. In addition to morphological malformations, also effects on larval swimming behavior, as indication for larval fitness, and biochemical endpoints were included. One important biomarker of exposure, which is typically chosen and highly sensitive toward petroleum product exposure, is the induction of the metabolic enzyme CYP1A (Sanni et al. 2017; van der Oost et al. 2003). Thus, the 7-ethoxyresorufin-O-deethylase (EROD) activity was examined as a very sensitive marker for the CYP system in fish (van der Oost et al. 2003). Previous studies have demonstrated that swimming behavior is altered in oil WAF–exposed larval fish (Johann et al. 2020; Mager et al. 2014; Perrichon et al. 2016) already at low exposure concentrations, thus indicating this endpoint to be a sensitive biomarker for changes in general fitness of petroleum compound-exposed fish larvae. Since alterations in swimming behavior can indicate a neurotoxic potential of toxicants (Legradi et al. 2018), in the present study, the activity of acetylcholinesterase (AChE) was additionally selected as a mechanism-specific biomarker for neurotoxicity.

In combination with other findings from the large-scale field experiment in Kangerluarsoruseq in 2017, the results of the present study aimed at contributing to the environmental evaluation of toxic effects induced by ISB residues of oil.

## Material and methods

### Sample background

The heavy fuel oil IFO 180 (Polaroil, Greenland) was used in the current study. All experiments presented were performed with oil from one batch. IFO 180 is a bunker oil characterized by a high viscosity with low amounts of volatile hydrocarbons (King et al. 2017). The ISB residues were generated during a large-scale ISB field experiment in a bay of Kangerluarsoruseq, Greenland. One thousand liters of IFO 180 was released on the water surface in a fire-resistant boom (Desmi Pyroboom, Desmi A/S, Denmark). After ignition, the oil burned for approximately 40 min. A video of the ISB experiment is available online (). After the burning, a trawl skimmer (Desmi A/S, Denmark) was used to collect the burn residue. After collection, aliquots of the sticky and highly viscous ISB residues were collected on absorbant oil-wetting cloth (3M™ Oil Sorbents) in RILSAN bags for shipping. Immediately after ISB residues arrived in the laboratory, burn residues were scraped from collecting bags using inert spatula that had been rinsed with solvents (acetone, pentane) and aliquoted to avoid multiple thawing and re-freezing. Aliquots were stored at – 20 °C until further usage.

In order to investigate the influence of chemical dispersants, the third-generation dispersant Finasol OSR 52® (Total Special Fluids, France) was used. This dispersant contains > 30 % non-ionic and 15–30% anionic surfactants. Details can be found in the corresponding safety datasheet (SDS no. 30034 2013).

### Preparation of water-accommodated fractions (WAFs)

Water-accommodated fractions (WAFs) were prepared according to Singer et al. (2000). For the IFO 180 and ISB residues of IFO 180 exposure experiments, low-energy water-accommodated fractions (LEWAFs) were prepared. By the application of the dispersant Finasol OSR 52^®^, so-called chemically enhanced water-accommodated fractions of the initial oil (CEWAF) were prepared. Briefly, WAFs were prepared in aspirator glass flasks (500 mL). For the preparation of initial oil LEWAF, IFO 180 was carefully added to water surface of 300 mL fish medium at an oil-to-water ratio of 1:50 (w/v). To prepare ISB residue LEWAFs, the in situ collected and frozen sticky burn residue aliquots were thawed and applied to the water surface comparable with initial oil LEWAF (1:50, w/v). CEWAF was prepared by adding IFO 180 and the dispersant (1:10, w/w) to the water surface to reach a dispersed oil-to-water ratio of 1:200 (w/v). The LEWAF setups were carefully stirred with low energy avoiding a vortex while the CEWAF was stirred at higher stirring speeds (25% depth vortex in water column). WAFs were incubated while stirring at 10 °C for 40 h followed by 1 h settling time. Afterward, water fractions were carefully drained off. Different dilutions prepared from the 100% stock solutions were warmed up to 26 °C before embryos were exposed to the samples.

### Zebrafish maintenance and egg production

Wild-type zebrafish of the West Aquarium strain (Bad Lauterburg, Germany) from the facilities of the Institute for Environmental Research, RWTH Aachen University, were used. Breeding groups of 100–120 adult zebrafish from 1 to 3 years of age were kept in 170-L tanks of a flow-through system with an automatic water exchange rate of 40% per week. Tank water was cleaned through a biological filter and UV light. Fishes were fed with dry flakes (TetraMin^®^, Tetra GmbH, Germany) and larvae of *Artemia* spec. (JBL GmbH & Co. KG, Germany). A constant day-night rhythm (14:10) and temperature (26 ± 1 °C) was maintained. Spawning took place from 30 min after the onset of light. Fertilized eggs were collected 2 h after the onset of spawning.

### Fish embryo acute toxicity test

The acute toxic and teratogenic effects induced by the different WAF treatments were investigated using the prolonged fish acute embryo toxicity test up to a maximum developmental stage of 120 h post fertilization (hpf). The test was performed according to OECD guideline 236 (OECD 2013) with minor modifications due to sample type specifications (details described below). All experiments were terminated with the final measurement shortly before 120 hpf, so that no animal ethics test approval was required. Zebrafish embryos and larvae below 120 hpf are not protected animal stages according to EU Directive 2010/63/EU (European Union 2010), see also Strähle et al. (2012), TierSchG (Tierschutzgesetz), and the respective regulation TierSchVerV (Tierschutz-Versuchstierverordnung). After termination, larvae were euthanized by prolonged immersion in a benzocaine ethanol solution.

Briefly, 20 embryos per treatment concentration were transferred to sample dilutions shortly after fertilization. Embryos were incubated at 26 °C using a semi-static approach with periodic medium exchange (every 24 h). WAFs for medium exchange were prepared fresh daily. Artificial fish medium was prepared, aerated, and warmed up 1 day before using. The pH of all media was adjusted between 7.0 and 8.0.

Embryos were exposed in air-sealed 10-mL glass vials (5 embryos/vial) with sparse headspace to minimize the evaporation of volatile WAF compounds. In each experiment, negative (artificial water) and positive controls (3,4-dichloranilin, 4 mg L^−1^) were included to verify test validity. Embryos were inspected for lethal and sublethal effects every 24 h. Additionally, medium pH was monitored every 24 h. An experiment was classified valid if no more than 10% of negative control and at least 30% of positive control eggs showed lethal effects according to the OECD 236 guideline. All data presented met the validity criteria according to the guideline. Concentration-response curves were established according to details described in “Data analysis.”

### Light/dark transition test—larval swimming behavior

The exposure of zebrafish embryos was performed according to the exposure scenario described for the fish embryo acute toxicity test with the exception of 10 instead of 5 embryos per glass vial. In order to focus on the specific toxicity of the tested WAFs and to avoid masking effects by morphological deformations, embryos were exposed to sublethal effect concentrations (EC_10_–EC_20_). At 96 hpf, larval swimming behavior alterations were investigated using a light/dark transition test. This behavior test allows to monitor for dynamic behavioral responses (hyperactivity, hypoactivity, immobility), and it is sensitive to neuroactive chemical compounds (Ali et al. 2012; Legradi et al. 2015). The swimming performance of WAF-treated larvae as well as unexposed larvae was tracked during the experiment. Sixteen to 20 zebrafish larvae per treatment were transferred individually into one well of a 96-well plate and exposed to 2 cycles of alternating light (10 min) and dark (4 min) periods after an initial acclimatization time of 10 min in light conditions. The behavioral test was conducted using a DanioVision observation chamber and EthoVision tracking software (Noldus, The Netherlands).

## Biomarkers of xenobiotic metabolism and neurotoxicity

In another independent experimental setup, 40 embryos per treatment concentration were exposed to different sublethal effect concentrations as described before for the fish embryo acute toxicity test. Exposure concentrations for both biomarkers (AChE, EROD) were selected based on sublethal effect concentrations (below EC_50_) in order to avoid masking of secondary toxicity. At 96 and 120 hpf, the activity of the enzymes EROD and AChE in unexposed and WAF-treated zebrafish larvae was measured. Pre-hatching time windows were not included since the biomarker responses are highly variable due to embryonic developmental processes (Bräunig et al. 2015; Meyer-Alert et al. 2018). In total, 40 embryos per treatment concentration were pooled after the exposure, anesthetized using a cold solution of saturated benzocaine and washed twice with cold phosphate buffer saline (PBS, Sigma-Aldrich). Anesthetized larvae were quickly transferred to 1.5- mL tubes, and excess solution was replaced by 700 μl of phosphate buffer (1.8 L 0.1 M Na_2_HPO_4_ adjusted with 0.5 L 0.1 M NaH_2_PO_4_ to pH 7.8). Larvae were immediately shock frozen in liquid nitrogen and stored at − 80 °C until further use.

### Preparation of fish homogenates

For enzyme activity measurement, larvae in buffer were carefully thawed on ice and homogenized (VDI 12, VWR International GmbH, Germany) for 10 s. Afterward, homogenates were centrifuged at 10,000×*g* and 4 °C for 15 min. The supernatant was immediately transferred to new tubes and placed on ice. Both EROD and AChE were measured successively in order to avoid re-freezing and thawing.

### 7-Ethoxyresorufin-O-deethylase (EROD) activity in zebrafish larvae

The fish embryo EROD assay to investigate the catalytic induction of the fish cytochrome P450 system indicating a phase I metabolism was performed according to Schiwy et al. (2014) with modifications regarding egg number and quantification method (kinetic instead of two points measurement). The reference calibration series of resorufin (0.5 μM in 0.1 M Na_2_PO_4_ buffer) was prepared in duplicates in a 96-well plate (100 μL) using a 1:2 dilution series. Sample supernatants of WAF treatment and negative control larvae were added in triplicates to the plate (100 μL). Afterward, 100 μL of the substrate 7-ethoxyresorufin (2.4 μM) was added to each well followed by 10 min incubation at 26 °C in darkness. Shortly before kinetic measurement of fluorescence for 25 min (step 1: kinetic cycles: 15, interval time: 20 s, step 2: kinetic cycle: 30, interval time: 40 s) in a microplate reader (Infinite® M 200, Tecan Group, Switzerland), 50 μL NADPH (3.35 mM) was added to initiate the enzymatic reaction. Substrate deethylation was determined by measuring the formed resorufin at 540 nm excitation and 590 nm emission wavelength. Quantification of EROD activity was performed based on the resorufin calibration series and expressed in pmol resorufin mg^−1^ min^−1^.

### Acetylcholinesterase (AChE) activity in zebrafish larvae

The AChE activity as a surrogate endpoint for neurotoxicity was measured according to the initial protocol established by Ellman et al. (1961) with modifications according to Velki et al. (2017) regarding adaptions to a 96-well plate format. A total of 7.5 μL sample supernatant and 180 μL sodium phosphate buffer (0.1 M, pH 7.8), 10 μL 5,5′-dithiobis-2-nitrobenzoic acid (DTNB 1.6 mM), and 10 μL acetylcholine iodide (156 mM) were added to a 96-well plate. The increase in absorbance was immediately measured in triplicates at 412 nm for 10 min in 10-s intervals using a microplate reader (Infinite^®^ M 200, Tecan Group, Switzerland).

Resulting data were controlled for linearity in absorbance increase (*R*^2^ ≥ 0.98) and minimum increase of absorbance over time (Δt3min ≥ 0.1). Only data fulfilling these criteria were used for further calculations. Enzymatic activity was calculated as nmol AChE min^−1^ mg^−1^. For the calculations, the molar extinction coefficient of 13,600 M^−1^ cm^−1^ was used.

### Protein measurement

Whole protein of each sample was measured in parallel using a bicinchoninic acid assay kit (Sigma Aldrich GmbH, Germany) according to the manufacturer’s instructions and quantified with a dilution series of bovine serum albumin (BSA) as an external standard (0.31–1 mg mL^−1^). For protein measurement, the sample supernatants were diluted 1:2.

## Chemical analysis of LEWAFs

A basic chemical analysis of 18 target PAHs, including 16 USEPA PAHs in native and burned LEWAF, was conducted using solid-phase micro extraction (SPME) (Potter and Pawliszyn 1994). Target PAHs were selected according to relevant quantities from a detailed chemical profile of IFO 180 (see SI Tables S2 and S3) and the relevance from toxicological perspective. Methods of the detailed chemical profile can be found in the SI. The extraction method was not applied to CEWAF exposure as the dispersed oil droplets would interfere with the loading rates. The stability of the target PAH composition in LEWAFs during the experiment with periodic medium exchange (every 24 h) has been shown in preliminary tests. Thirty-micrometer silicone-coated fibers (Supelco, Sigma-Aldrich Corp) were applied to extract target PAHs directly from the exposure stock solutions after 40 h incubation and 1 h settling time. Perdeuterated internal standard PAHs were added to the samples prior to extraction. External (S-4008-100-T) and perdeuterated internal standards (S-4124-200-T) were purchased from Chiron (Chiron AS, Trondheim, Norway). SPME fibers were extracted for 2 h to enable quantification of low concentrations of target PAHs in the test medium. Loaded SPME fibers were analyzed using an Agilent Technologies GC system (7890 A GC System and 5975 C inert XL MSD with Triple-Axis-Detector, Agilent Technologies Deutschland GmbH).

### Data analysis

All WAF concentrations were presented as dilution of the stock solution (% of stock) as the present chemical analysis was limited and hence no defined concentration, e.g., in ∑PAH, would represent the real scenario. For the acute fish embryo toxicity test, concentration-response curves were established using the software GraphPad Prism (version 6, San Diego, USA). EC_*x*_ (concentrations inducing *x* % of sublethal and lethal effects) values were calculated based on a 4-parameter non-linear regression model with top and bottom set to 100 and 0, respectively, in GraphPad Prism (Equation: *Y* = 100 / (1 + 10^((LogEC50-X) * HillSlope)).

Data processing of results from swimming alteration and enzyme activity measurements was conducted in spreadsheets (Microsoft Excel 2016). In detail, the mean distance moved of 16–20 larvae per treatment was used for further evaluation of swimming patterns. In total, 3 independent experiments were performed, and hence, data were plotted and analyzed as mean (3 experiments) of mean (20 individuals per experiment) distance moved per interval. Specific enzymatic activity was normalized to the mean activity of the untreated control in order to guarantee comparability within the study and with previous studies.

Statistical analyses were performed in SigmaPlot (Version 12.5, Systat Software, 2007). Data were analyzed for normal distribution (Shapiro-Wilk test) and homoscedasticity (Levene test) and then further investigated for statistical significance, with difference compared with untreated control. Data fulfilling both criteria were analyzed using one-way analysis of variance (ANOVA) with Dunnett’s post hoc test (*p* < 0.05). The non-parametric Kruskal-Wallis ANOVA on ranks with Dunn’s post hoc test was used for data that were not normally distributed and/or did not show homoscedasticity

## Results

### Acute fish embryo toxicity

Concentration-related increases in sublethal and lethal effects were observed for WAFs from initial IFO 180, ISB residues of IFO 180, and chemically dispersed IFO 180. At the termination of the test (120 hpf), all zebrafish larvae exposed to 66.7 % of the initial IFO 180 LEWAF stock (1:50) were defined as dead according to the lethality criteria defined by the OECD guideline 236. In contrast, the ISB residue LEWAF exposure did not result in 100 % mortality even at the highest test concentration of the undiluted stock (1:50). The CEWAF stock (1:200) was more toxic to the zebrafish embryos. Even concentrations of 12.5 % of stock led to 100 % mortality at 120 hpf. Hence, based on the concentration-response curves (see SI, Figure S1), the calculated 50 % effect concentrations (EC_50_) decreased from exposure to WAFs of the ISB residue, via the initial IFO 180 to chemically dispersed IFO 180 (Table 1).

**Table 1:** Effect concentrations (EC_*x*_) in the acute fish embryo toxicity test with zebrafish larvae (120 hpf) exposed to WAFs of initial, burned, and dispersed IFO 180. EC values were calculated by sigmoidal concentration-response curves fitted in Prism 6 using the 4-parameter non-linear regression model with top and bottom variables set to 100 and 0, respectively (*n* = 3). 95 % confidence interval (CI) was included. ND indicates that no lower/upper limit of CI could have been estimated by the regression analysis.

	EC_10_ (% of stock)	95 % CI	EC_20_ (% of stock)	95 % CI	EC_50_ (% of stock)	95 % CI
IFO 180 LEWAF	16.5	ND	18.9	ND–21.38	23.7	21.60–25.69
burned IFO 180 LEWAF	13.4	(ND–24.60)	20.3	ND–30.41	41.4	30.34–54.70
IFO 180 CEWAF	1.9	(1.38–2.68)	2.3	1.65–2.83	2.9	2.30–3.37

**Fig. 1:**
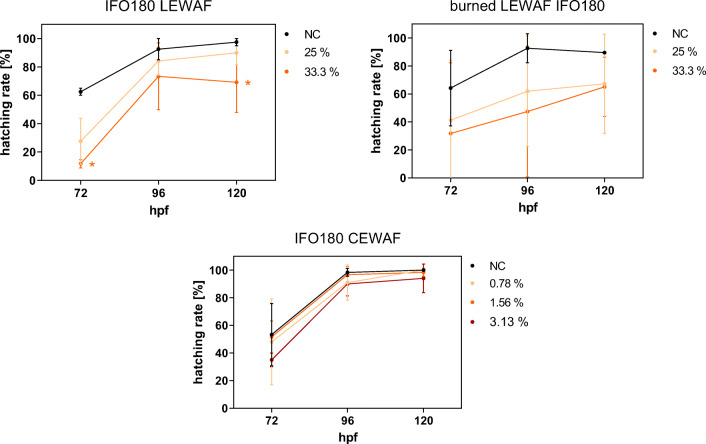
Hatching success over time of control and WAF-exposed zebrafish larvae. Hatching success was calculated for sublethal effect concentrations (% of WAF stock) and unexposed negative control (NC) during the acute fish embryo toxicity test. Points and error bars denote the mean and standard deviation of 3 individual experiments. Asterisks indicate statistically significant difference from NC (Kruskal-Wallis one-way ANOVA on ranks with Dunn’s post hoc test, *p* < 0.05)

The most prominent morphological effects observed in zebrafish embryos exposed to all WAF treatments were heart deformation and yolk sac or pericardial edema in combination with bradycardia and blood circulatory interruptions (Table 2, see also SI Table S1: all morphological effects across all time points of observation). Furthermore, spine deformations were observed frequently. The hatching success was not affected in sublethal effect concentrations of the chemically dispersed IFO 180 CEWAF, but sublethal LEWAF concentrations of both IFO 180 and burned IFO 180 induced a delayed hatching compared with control embryos. While > 90 % of control embryos were hatched after 96 hpf, the LEWAF-exposed embryos reached a maximum hatching rate of 60–80 % at 120 hpf (Fig. 1). However, even for LEWAF-exposed embryos, hatching increased over time.

**Table 2:** Morphological sublethal effects in 120 hpf zebrafish embryos exposed to a dilution series of WAFs from initial, burned, and dispersed IFO 180. Data represent mean and standard deviation (*n* = 3) of % effects observed during acute fish embryo toxicity test. Exposure concentrations were based on preliminary experiments, which identified an exposure range resulting in 0–100 % effects. Detailed sublethal and lethal effects across all exposure dilutions and time points of observation (48–120 hpf) can be found in the SI (Table S1)

		% effects				
IFO 180 LEWAF	Exposure dilution (%) of stock	66.7	50	33.3	25.5	16.7
	Edema (heart)	28.3 ± 10.4	45.0 ± 27.8	46.7 ± 18.9	15.0 ± 13.2	6.7 ± 11.5
	Edema (yolk sac)	11.7 ± 16.1	1.7 ± 2.9	-	1.7 ± 2.9	-
	Slow heartbeat	6.7± 7.6	5.0 ± 8.7	11.7 ± 12.6	8.3 ± 5.8	3.3 ± 2.9
	Heart deformation	10.0 ± 5.0	25.0 ± 25.0	18.3 ± 18.9	11.7 ± 5.8	1.7 ± 2.9
	Slow blood flow	-	-	1.7 ± 2.9	-	-
	Blood congestion	1.7 ± 2.9	1.7 ± 2.9	5.0 ± 8.7	16.7 ± 24.7	-
	Spine deformation	21.7± 15.3	28.3 ± 27.5	31.7± 32.5	6.7 ± 11.5	6.7 ± 7.6
Burn residue IFO 180 LEWAF	Exposure dilution [%] of stock	100	66.7	50	33.3	25.5
	Edema (heart)	57.5 ± 24.7	52.5 ± 3.5	32.5 ± 38.9	20.0 ± 7.1	2.5 ± 3.5
	Edema (yolk sac)	27.5 ± 3.5	2.5 ± 3.5	-	12.5 ± 10.6	-
	Slow heartbeat	7.5 ± 10.6	10.0 ± 7.1	12.5 ± 17.7	5.0 ± 0.0	2.5 ± 3.5
	Heart deformation	22.5 ± 10.6	15.0 ± 14.1	20.0 ± 28.3	7.5 ± 10.6	-
	Slow blood flow	-	-	2.5 ± 3.5	-	-
	Blood congestion	-	-	-	2.5 ± 3.5	-
	Spine deformation	17.5 ± 24.7	30.0 ± 42.4	32.5 ± 46.0	2.5 ± 3.5	5.0 ± 0.0
IFO 180 CEWAF	Exposure dilution [%] of stock	12.5	6.3	4.2	3.1	1.6
	Edema (heart)	-	-	-	-	-
	Edema (yolk sac)	-	10.0 ± 13.2	50.0 ± 50.0	10.0 ± 17.3	1.7 ± 2.9
	Slow heartbeat	-	13.3 ± 15.3	35.0 ± 26.5	-	-
	Heart deformation	-	13.3 ± 15.3	75.0 ± 25.0	45.0 ± 36.1	6.7 ± 7.6
	Slow blood flow	-	-	5.0 ± 5.0	3.3 ± 5.8	-
	Blood congestion	-	-	1.7 ± 2.9	1.7 ± 2.9	-
	Spine deformation	-	20.0 ± 8.7	58.3 ± 5.8	33.3 ± 38.2	3.3 ± 2.9

### Alterations in larval swimming behavior

**Fig. 2:**
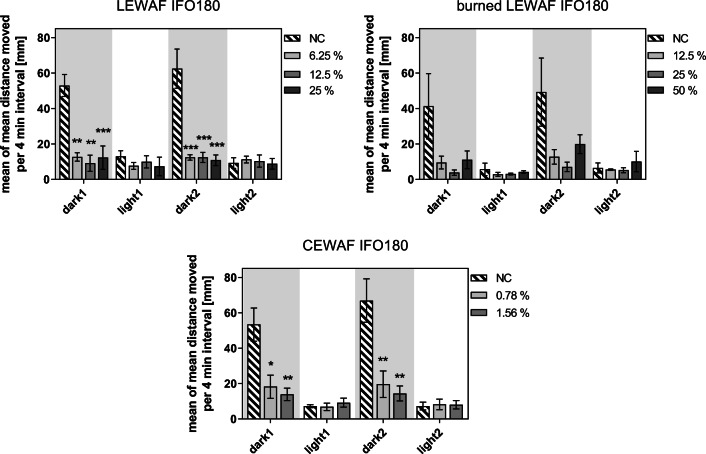
Swimming behavior of zebrafish larvae (96 hpf) exposed to sublethal effect concentrations of WAFs from initial, burned, and dispersed IFO 180 in a light/dark transition test. Bars and error bars represent the mean distance moved of 16–20 larvae per experiment further averaged over the 3 (initial) or 4 (burned, dispersed) independent experiments with standard error. Statistically significant difference of treatments compared to negative control (NC) was evaluated using one-way ANOVA with Dunnett’s post hoc test (*p* < 0.05). Non-parametric Kruskal-Wallis ANOVA on ranks was used for burned LEWAF analysis

Control zebrafish larvae showed the expected swimming behavior of high activity during the dark and low activity during the light phase in the light/dark transition test (for graphs on distance moved over time see SI Figure S2). Independent of the concentration, all zebrafish larvae exposed to the initial IFO 180, ISB residue of IFO 180, and chemically dispersed IFO 180 displayed altered swimming behavior during the dark stimulus compared with the unexposed control. While the exposed larvae showed a baseline swimming activity comparable to the control during the light phase, their swimming activity was significantly reduced during the sudden dark period (Fig. 2). However, different treatments induced slight deviating responses during dark periods. Larvae of the initial IFO 180 LEWAF treatment did not change the baseline swimming activity independent of the light/dark alterations. ISB residue LEWAF- and CEWAF-exposed larvae slightly increased their swimming activity when the light was switched off. However, differences were not statistically significant. A direct comparison of swimming behavior alterations between compliant nominal initial and ISB residue LEWAF exposure concentrations (12.5 and 25 % of stock) can be found in the SI (Figure S3).

## Biomarkers of xenobiotic metabolism and neurotoxicity

**Fig. 3:**
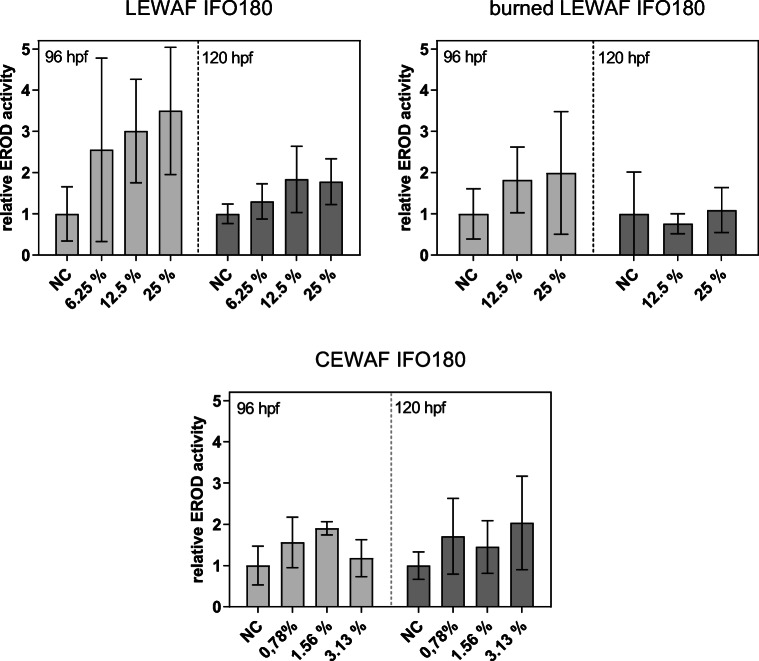
Relative 7-ethoxyresorufin-O-**d**eethylase (EROD) activity in zebrafish larvae exposed to WAF dilutions of initial, burned, and dispersed IFO 180. Specific EROD induction was normalized to the mean specific activity of the unexposed negative control (NC). Bars and error bars represent mean and standard deviation of the normalized activity of 3 independent experiments. After verifying normal distribution and equal variance, no statistically significant differences compared with NC was detected using one-way ANOVA with Dunnett’s post hoc test

While for both initial and ISB residue IFO 180 LEWAF a concentration-dependent increase in EROD activity was observed, no clear trend was found for the chemically dispersed IFO 180 (Fig. 3). The initial IFO 180 LEWAF exposure resulted in the highest EROD activity with a maximum induction of 3.5-fold compared with negative control. The maximum normalized EROD induction of ISB residue LEWAF- and CEWAF-exposed larvae were 1.9-fold and 2-fold, respectively. Both LEWAFs from initial and ISB residue IFO 180 led to a higher EROD activity in 96 hpf larvae than in 120 hpf larvae. A trend for a reduced EROD activity in larvae exposed to ISB residue compared with larvae exposed to initial oil was observed. In this respect, a 0.8-fold (96 hpf) and 2-fold (120 hpf) higher EROD induction in larvae exposed to initial LEWAF was observed for comparable nominal WAF dilutions (graphs for direct comparison see SI Figure S4).

**Fig. 4:**
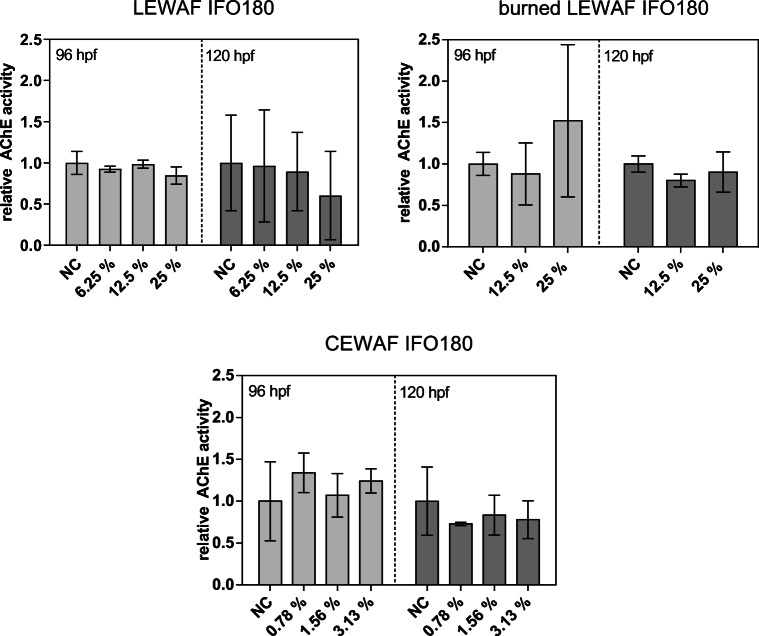
Relative acetylcholinesterase (AChE) activity in zebrafish larvae exposed to WAF dilutions of initial, burned, and dispersed IFO 180. Specific AChE induction was normalized to the mean specific activity of the unexposed negative control (NC). Bars and error bars represent mean and standard deviation of normalized activity of 3 independent experiments. After verifying normal distribution and equal variance, no statistically significant differences compared with NC were detected using one-way ANOVA with Dunnett’s post hoc test

As a biomarker of neurotoxicity, the inhibition of the acetylcholinesterase (AChE) was investigated. In contrast to clear effects in the EROD assay, the AChE activity following exposures to the LEWAFs and the CEWAF of the IFO180 did not differ from that in the non-exposed control larvae independent of the larval developmental stage (96 and 120 hpf) (Fig. 4). Nevertheless, the highest exposure concentration of the initial IFO 180 LEWAF led to a decrease in AChE activity (60 % of control) in 120 hpf larvae, which was not statistically significant. For the AChE activity, differences between both LEWAF treatments were less clear than for EROD activity, showing a stronger enzyme inhibition induced by the initial LEWAF only in 120 hpf zebrafish larvae (SI, Figure 4).

### Identification of target PAHs in LEWAFs

Naphthalene, fluorine, and phenanthrene were the most dominant compounds among the investigated target PAHs and detected in the μg L^−1^ range (Table 3). The remaining PAHs were detected in concentrations < 1 μg L^−1^. PAH concentrations in the LEWAF from ISB residues (total PAH 52.2 μg L^−1^) were substantially reduced compared with the initial LEWAF (total PAH 184.8 μg L^−1^). The dissolved fractions of the 3 most prominent target PAHs were reduced by 62% (fluorene), 66% (phenanthrene), and 73% (naphthalene), respectively. Importantly, the difference in total PAH concentration was mainly related to the semi-volatile naphthalene. Without naphthalene, total PAH in both initial and burn residue LEWAF were in a comparable range of 15.6 and 6.4 μg L^−1^.

**Table 3:** Target PAHs in LEWAF stocks (1:50) of initial and burn residues of IFO 180. LEWAF stocks were prepared in artificial fish medium (40 h mixing). PAHs were extracted from medium using solid-phase micro extraction (SPME) for 2 h and analyzed using GC-MS

Target compound	Initial LEWAF IFO 180 (μg L^−1^)	Burned LEWAF IFO 180 (μg L^−1^)
Naphthalene	169.16	45.78
Fluorene	4.51	1.73
Phenanthrene	8.38	2.86
Anthracene	0.61	0.25
Fluoranthene	0.13	0.09
Pyrene	0.42	0.20
11h-benzo[a]fluorene	0.33	0.25
11h-benzo[b]fluorene	0.23	0.19
Benzo[a]anthracene	0.14	0.15
Chrysene	0.42	0.35
Benzo[b]fluoranthene	0.10	0.08
Benzo[k]fluoranthene	0.12	0.09
Benzo[a]pyrene	0.14	0.11
Benzo[e]pyrene	0.08	0.08
Indeno[1,2,3-c,d]pyrene	Not detected	Not detected
Dibenz[a,h]anthracene	Not detected	Not detected
Benzo[g,h,i]perylene	Not detected	Not detected
Dibenzo[a,e]pyrene	Not detected	Not detected
Σ PAH	184.77	52.21
Σ PAH (without naphthalene)	15.61	6.43

## Discussion

### Environmental effect assessment of in situ burning in comparison to chemical dispersion

On the effect level, ISB did not increase the toxicity to zebrafish early life stages compared with the initial LEWAF. Though not significant, the results of the present study indicate a trend of a reduced embryo toxicity for LEWAFs from ISB residues compared with untreated IFO 180 LEWAFs.

The exposure to CEWAF (dispersed oil) induced much higher acute embryo toxicity compared with the initial or burn residue LEWAFs. However, when tested in corresponding effect-concentrations in the range of EC_10_–EC_20_, the resulting effects of CEWAF exposure on hatching success, swimming behavior, or enzyme activities were comparable with or even below the effects observed for both LEWAF treatments. This indicates that a higher CEWAF toxicity is rather related to the dispersion effect changing the hydrocarbon partitioning between the water/oil phase than to a toxicity of the dispersant itself, which is in accordance with the current understanding of dispersant effects (Carls et al. 2008; Redman and Parkerton 2015). Increased relative PAH concentrations in the aqueous solution of WAFs after the application of a dispersant have been observed in several studies (Cohen 2001; Couillard et al. 2005; Hook and Osborn 2012). The increase of dissolved oil compounds in the CEWAF indicates that the initial LEWAF system of the present study was not in a stable state of equilibrium after 40 h of mixing, in which droplet formation should not change the compound partitioning between oil and water phase approximately calculated based on Raoult’s law (Lee et al. 1992). Nonetheless, previous experiments with Finasol OSR51^®^ showed acute toxicity to zebrafish embryos by the dispersant itself, especially after hatching (Johann et al. 2020). Hence, the dispersant might also contribute to the CEWAF toxicity to a certain extent.

Compared with detailed discussions on the role of dispersants in petroleum product toxicity (Prince 2015), a limited number of laboratory experiments investigating the toxic effects of burn residues appear to be available as peer-reviewed scientific articles. In fact, most studies so far tested the water phase underlying an ignited oil slick directly after laboratory ISB experiments (Bender et al. 2018; Cohen et al. 2006; Faksness et al. 2012), which limits the comparability to the present approach (due to weathering in the field etc.). Furthermore, some studies do not compare with WAF samples from untreated petroleum products or other oil spill response techniques, which limits the evaluation of the ISB residue toxicity. However, in general, a low acute toxicity of burn residue WAFs to marine invertebrate species has been observed (Faksness et al. 2012). Besides invertebrate species, also different fish species have been exposed to WAFs from ISB experiments (Bender et al. 2018; Cohen 2001; Cohen et al. 2005). The studies mainly have found a low toxic potential (Cohen et al. 2005). However, also delayed effects after a recovery phase such as reproductive impact have been observed in fish that have been exposed to burn residues for a short period (Bender et al. 2018).

Using a comparable experimental setup as in the present study, Blenkinsopp et al. (1996) focused on the toxicity of residues from the Newfoundland offshore burn experiment by preparing WAFs from the field residue samples and the (weathered) crude oil under laboratory conditions. The experiments, which included freshwater and marine experiments with rainbow trout (*Oncorhynchus mykiss*), stickleback (*Gasterosteus aculeatus*), and sea urchins (*Lytechinus pictus*), revealed no toxicity in any of the tests (Blenkinsopp et al. 1996).

For the interpretation and extrapolation of ISB toxicity assessment from the present study, limitations have to be considered. The WAFs in the present study were prepared at 10 °C and further tested at 26 °C, which do not represent arctic conditions. Hence, the influence of extreme artic environmental conditions, e.g., low seawater temperature, and long hours of sunlight, affecting the dynamics, behavior, and potentially composition of dissolved hydrocarbons in the water column, has to be considered for the effect extrapolation (Wegeberg et al. 2018b). Brown et al. (2016) showed that the hydrocarbon partitioning in WAFs in cold seawater conditions is strongly temperature dependent. Even though it was expected to be less influenced by temperature changes due to low amounts of evaporative low-molecular-weight hydrocarbons, Brown et al. (2016) found that the dissolved fraction of a heavy fuel oil such as IFO 180 increased with increasing temperature from arctic (0 °C) to subarctic (5 °C) marine conditions. In contrast to the distillates tested in that study, the IFO 180 depletion rate did not seem to be changed over time in the present study. Though to a reduced extent, also water salinity changes hydrocarbon partitioning between the oil and water interphase. For several PAHs, a decreased solubility with increasing salinity has been reported (Eganhouse and Calder 1976; Saranjampour et al. 2017; Whitehouse 1984). Hence, the present study preparing WAFs at 10 °C under freshwater conditions using semi-static closed exposure instead of open static exposure scenario can be interpreted as a worst-case scenario.

Overall, the practical application of ISB or chemical dispersants is challenging for heavy fuel oils such as IFO 180 due to the physical chemical properties, which might influence the decision about a selected response measure. In this context, e.g., relatively high minimum slick thickness and heat flux are necessary for a successful ignition and a high burning effectiveness (Fritt-Rasmussen 2010). Highly viscous oils need a higher amount of surfactants to be successfully dispersed into the water column. In addition, progressing weathering reduces the effectiveness of both methods (Fritt-Rasmussen et al. 2012).

### Acute toxicity of heavy fuel oil compounds in fish embryos

Compared with crude oils and refined distillates, the dissolved hydrocarbon content of IFO 180 in the water column is reported to be relatively low (Brown et al. 2016) due to the chemical composition with much higher concentrations of high-molecular-weight compounds (e.g., 3+-ring PAHs, resins, asphaltenes) relative to low-molecular-weight hydrocarbons (1–2 ring aromatics) (Redman and Parkerton 2015). This might lead to the assumption that after an oil spill, the exposure of the pelagic community to water-soluble fractions of heavy fuel oils, and hence the ecotoxicological risk, is limited compared with lighter refined products (Fritt-Rasmussen et al. 2018). Nonetheless, the present study identified phenotypic embryonic malformations when exposed to WAFs of IFO 180, which is in accordance with tests on a variety of other oil types across different freshwater and marine species (for review see Hodson 2017; Incardona 2017). Moreover, the chemical profile of the IFO 180 (see SI Tables S2 and S3) as well as the LEWAF composition of the tested IFO 180 indicated that the presence of toxicologically relevant concentrations of PAHs was in comparable ranges with lighter petroleum products (e.g., Hodson 2017). However, it has to be considered that the exposure concentrations may have exceeded environmentally relevant concentrations occurring after an oil spill. In the water column, total PAH concentrations have been reported to be 5–10 times below the concentrations detected in the present WAF stocks (Echols et al. 2015). Hence, the exposure concentrations tested in the current study assess a worst-case scenario to identify potential differences between the response measures.

In oil toxicity assessment, one major challenge is the explanation of observed effects by the chemical composition of the oil type. To a certain extent, ISB might change the chemical profile of a spilled oil, which might lead to modified toxicity. However, Faksness et al. (2012) did not report differences in the chemical oil profile prior and after the burning. Nonetheless, focusing on the water phase under untreated or burned oil slick, the present findings of reduced concentrations of target PAHs in burn residue LEWAF compared with untreated oil LEWAF is in agreement with results from previous studies (Bender et al. 2018; Blenkinsopp et al. 1996). However, it has to be considered that the decrease of target PAHs was mainly related to the semi-volatile naphthalene. In addition, other compounds of lower concentration may possess different fate and effect patterns and as such represent different physical-chemical classes that are important in different time aspects of an oil spill (Kristensen et al. 2015). It has to be considered that from an analytical chemistry perspective, it cannot be excluded that due to reduced PAH compounds in ISB residues, the toxic potency is relatively increased in this approach.

The current understanding of acute embryo toxicity mainly identifies higher-molecular-weight target PAHs (e.g., phenanthrene) as strong toxicity drivers (Incardona et al. 2004, 2006; Le Bihanic et al. 2014). The role of the semi-volatile naphthalene, which was the dominant PAH in current analysis, is controversially discussed. Several studies found no contribution of naphthalene to adverse effects in embryos (Adams et al. 2014; Black et al. 1983; Incardona et al. 2004), while others reported a high (zebrafish) embryo toxicity in experiments using passive dosing approaches (Seiler et al. 2014). Overall, even though AhR-dependent or -independent modes of actions of selected PAHs are understood, and that still more than 25% of variations in toxicity due to complex mixture interactions remains unexplained (Hodson 2017). Our previous experience with identical analysis and experimental setups supports this conclusion, since biological effect gradients across different oil types could not be explained by corresponding chemical analysis of target PAHs (Johann et al. 2020).

Certainly, it has to be considered that the present analysis focused on a limited set of target PAHs. PAH derivatives, especially alkylated congeners, typically represent a high proportion of petroleum product compounds (Andersson and Achten 2015) and are known to induce adverse effects in early developmental stages (Adams et al. 2014; Bornstein et al. 2014; Martin et al. 2014; Scott et al. 2011). From the chemical profile, it is known that especially alkylated naphthalenes and phenantrenes are present in the IFO 180 and hence can be expected to be present in the WAFs, potentially inducing the observed effects. However, PAHs generally represent a fraction of up to a maximum of 60% in crude oils (Dupuis and Ucan-Marin 2015; Fingas 2011). Furthermore, recent work has found a significant contribution of mono-aromatics from crude oils to typical phenotypes of oil-exposed fish embryos (Sørensen et al. 2019). Even bulk hydrocarbon analysis such as total petroleum hydrocarbons (TPH), which are frequently applied in oil studies, do not enable a precise hazard assessment (Hansen et al. 2018; Redman et al. 2018; Redman and Parkerton 2015). Hence, some uncertainty for effect explanation remains even with target PAH analysis, highlighting the importance of both chemical and biological data for risk assessment.

### Biomarker responses in sublethal exposure concentrations

Alterations of enzyme activities as well as swimming behavior in fish early life stages exposed to petroleum products or relevant compounds such as PAHs have been investigated extensively (de Soysa et al. 2012; Pauka et al. 2011; Perrichon et al. 2016).

The EROD activity is a reliable biomarker for petroleum product exposure as several PAHs induce the upregulation of xenobiotic biotransformation enzymes such as cytochrome P450 (van der Oost et al. 2003). In general, a strong EROD activity observed in the present study is in agreement with current scientific knowledge on EROD induction. It has been observed in whole-specimen supernatant (Perrichon et al. 2016) or livers (Arukwe et al. 2008; Pauka et al. 2011) after the exposure to crude oils or refined petroleum products independent of the exposure scenario, fish developmental stages, and species (Adams et al. 2014; Couillard et al. 2005; Kennedy and Farrell 2006; Oliveira et al. 2007; Ramachandran et al. 2004).

Comparable with the present study, metabolic enzyme activities in juvenile fish exposed to initial, burned, and dispersed crude oil have been examined before (Cohen 2001; Cohen et al. 2005, 2006). Deviating from the present results on chemically dispersed heavy fuel oil IFO 180, chemical treatment of the crude oil treatment in the aforementioned studies induced stronger EROD activities than initial or burned samples (Cohen et al. 2005, 2006). This discrepancy might be related to the dispersion capability, which is higher in crude oils compared with heavy fuel oils due to their lower viscosity (Fritt-Rasmussen et al. 2018). Hence, the dissolved and particulate fractions of the crude oil might have been more bioavailable and thus induced stronger biomarker responses. Further, the authors did not identify differences in EROD activities between initial and burned crude oil WAFs (Cohen et al. 2006), which is also deviating from the present findings. However, in our opinion, a direct comparison of effects induced by the initial crude oil and corresponding ISB residues is limited due to deviating WAF preparation methods for those two samples. While crude oil WAF was mixed for 20 h, the water phase underlying the burn residue was used immediately following a 45-min (Cohen et al. 2005) or a 20-min (Cohen et al. 2006) burning. This might have led to different time windows for hydrocarbon partitioning into the water column.

Alterations of swimming patterns in fish species such as zebrafish, Japanese medaka (*Oryzias latipes*), or mahi-mahi (*Coryphaena hippurus*) exposed to petroleum WAF have focused on different endpoints such as response to touch stimulus (de Soysa et al. 2012), performance in swim chambers (Hicken et al. 2011; Mager et al. 2014), and locomotor activity with or without a light/dark challenge system (Le Bihanic et al. 2014; Perrichon et al. 2014, 2016). A broad range of WAF or PAH treatment-related effects, from increased (Le Bihanic et al. 2014) to decreased (Perrichon et al. 2014) swimming activity or anxiety-related shelter seeking behavior (Philibert et al. 2016) compared with control groups, have been reported. However, also no effects on swimming behavior induced by WAF exposure have been reported (Perrichon et al. 2016). While previous work (Johann et al. 2020) and the present study observed consistent reduced swimming activity (during darkness) independent of the oil type, Perrichon et al. (2016) showed deviating responses of zebrafish larvae exposed to different oil types (light Arabian crude oil and HFO). In that particular study, one oil type reduced swimming activity, while the other oil did not affect swimming behavior at all. Differences in the reported responses in the various above-cited studies, including the present study, might be related to differences in experimental setups and the unique character of the specific oil types.

In general, alterations in fish swimming behavior patterns have mainly been related to cardiotoxicity (Hicken et al. 2011; Mager et al. 2014). However, in the present study, the tested sublethal exposure concentrations did not induce heart deformations, edema, or blood circulatory interruptions, which would be indicative for cardiotoxic effects. However, recent work showed that the disruption of cellular calcium ion signaling in cardiomyocytes strongly contributes to cardiotoxic effects (Brette et al. 2014, 2017; Incardona 2017). Since these molecular mechanisms were not investigated in the present study, an overall cardiotoxicity cannot be excluded. Other causes for swimming behavior alterations such as neurotoxicity might also have resulted in the observed alterations. The inhibition of the AChE has been suggested as a biomarker for neurotoxicity in fish of different developmental stages (Soreq and Seidman 2001; Velki et al. 2017; Yen et al. 2011). In general, measuring the AChE inhibition as a biomarker for neurotoxicity is less applied in the field of petroleum-induced toxicity. Nonetheless, also petroleum compounds such as PAHs potentially inhibit the enzyme as indicated by experiments using *in vitro* approaches with isolated enzymes from fish (Jett et al. 1999), whole larvae tissue measurements (Kais et al. 2015), or adult brain homogenates (Jee and Kang 2003). However, deviating from the clear inhibitory effects on AChE caused by neurotoxic pesticides (Velki et al. 2017), ambiguous results were reported for inhibitory effects on AChE caused by PAHs or crude oils (Kais et al. 2015; Tang et al. 2003). In the present study, no correlation between AChE and swimming behavior was found. Also, previous studies reported rather poor correlations between swimming behavior alterations and AChE inhibition in zebrafish (Haverroth et al. 2015; Tilton et al. 2011), which might indicate that AChE is not a very sensitive biomarker for the detection of effects from PAHs.

In general, though, a neurotoxic effect in the present study cannot be excluded, since only one enzyme was investigated. It should be noted that recent studies focusing on the neuronal development in early life stages of fish indicated neurotoxic effects caused by petroleum hydrocarbons (Gao et al. 2015; Vignet et al. 2017; Xu et al. 2017), indicating the relevance of neurotoxicity as a relevant endpoint for petroleum toxicity research. However, our current findings support previous conclusions that AChE is not a key biomarker for neurotoxic endpoints in petroleum research. Additionally, the lacking responses of WAF-treated larvae to a dark stimulus might also be linked to other effects, such as impairments of the visual system. Oculotoxicity has recently been discussed as a major aspect of oil-related toxicity (Xu et al. 2016, 2017).

## Conclusion

In the present study, there were no overt differences in the acute toxicity of IFO 180 and burned IFO 180 residues to zebrafish embryos. In contrast, the acute toxicity was higher for the dispersed IFO 180, most likely due to increased bioavailability of oil constituents. Though differences were not significant, it can still be concluded that ISB residues from IFO 180 are not more acutely toxic to fish embryos than the untreated, un-weathered oil slick. The present study provides information on effect level using bioanalytical tools, which has the major advantage of not presupposing knowledge about contaminant details in complex environmental samples. Future studies should include detailed chemical profiles in order to link toxicity drivers to observed adverse effects.

From a superordinate perspective, it has to be considered that ISB does not appear to reduce the concentration of toxic oil compounds already partitioned into the water column before ignition. Therefore, the acute toxicity of an oil spill is likely not eliminated by ISB, but might be reduced due to removal of large oil volume by combustion.

It should be noted that the present study applied a laboratory model species, the zebrafish, to study acute toxicity and biomarker responses. Thus, studies in wild fish species, including arctic endemic species, and under environmental realistic conditions need to be performed to confirm or refute the present findings prior to concluding on the possible beneficial environmental outcomes of in situ burning of oil spills on fish populations.

The results of the present study provide insight into possible adverse effects of ISB residues on sensitive fish life stages and can support environmental assessment of this oil spill response measure in the, e.g., mitigation assessment (SIMA) and Environment and Oil Spill Response (EOS) tools.

## Supplementary Information

ESM 1 (DOCX 234 kb).

## Data Availability

The datasets used and/or analyzed during the current study are available from the corresponding author on reasonable request.

## References

[CR1] Adams J, Bornstein JM, Munno K, Hollebone B, King T, Brown RS, Hodson PV (2014). Identification of compounds in heavy fuel oil that are chronically toxic to rainbow trout embryos by effects-driven chemical fractionation. Environ Toxicol Chem.

[CR2] Ali S, Champagne DL, Richardson MK (2012) Behavioral profiling of zebrafish embryos exposed to a panel of 60 water-soluble compounds. Behav Brain Res 228:272–283. 10.1016/j.bbr.2011.11.020 10.1016/j.bbr.2011.11.020 22138507

[CR3] Andersson JT, Achten C (2015). Time to say goodbye to the 16 EPA PAHs? Toward an up-to-date use of PACs for environmental purposes. Polycycl Aromat Compd.

[CR4] Arukwe A, Nordtug T, Kortner TM, Mortensen AS, Brakstad OG (2008). Modulation of steroidogenesis and xenobiotic biotransformation responses in zebrafish (*Danio rerio*) exposed to water-soluble fraction of crude oil. Environ Res.

[CR5] Bejarano AC, Clark JR, Coelho GM (2014). Issues and challenges with oil toxicity data and implications for their use in decision making: a quantitative review. Environ Toxicol Chem.

[CR6] Bender ML, Frantzen M, Camus L, Le Floch S, Palerud J, Nahrgang J (2018). Effects of acute exposure to dispersed oil and burned oil residue on long-term survival, growth, and reproductive development in polar cod (*Boreogadus saida*). Mar Environ Res.

[CR7] Black JA, Birge WJ, Westerman AG, Francis PC (1983). Comparative aquatic toxicology of aromatic hydrocarbons. Toxicol Sci.

[CR8] Blenkinsopp SA, Sergy G, Doe K, Wohlgeschaffen G, Li K, Fingas M (1996). Toxicity of the weathered crude oil used at the Newfoundland offshore burn experiment (NOBE) and the resultant burn residue. Spill Sci Technol Bull.

[CR9] Bornstein JM, Adams J, Hollebone B, King T, Hodson PV, Brown RS (2014). Effects-driven chemical fractionation of heavy fuel oil to isolate compounds toxic to trout embryos. Environ Toxicol Chem.

[CR10] Bräunig J, Schiwy S, Broedel O, Müller Y, Frohme M, Hollert H, Keiter SH (2015). Time-dependent expression and activity of cytochrome P450 1s in early life-stages of the zebrafish (*Danio rerio*). Environ Sci Pollut Res.

[CR11] Brette F, Cros C, Machado B, Incardona JP, Scholz NL, Block BA (2014) Crude oil impairs cardiac excitation-contraction coupling in fish. Biophys J 106:732a. 10.1126/science.1242747 10.1126/science.1242747 24531969

[CR12] Brette F, Shiels HA, Galli GLJ, Cros C, Incardona JP, Scholz NL, Block BA (2017). A novel cardiotoxic mechanism for a pervasive global pollutant. Sci Rep.

[CR13] Brown KE, King CK, Kotzakoulakis K, George SC, Harrison PL (2016). Assessing fuel spill risks in polar waters: temporal dynamics and behaviour of hydrocarbons from Antarctic diesel, marine gas oil and residual fuel oil. Mar Pollut Bull.

[CR14] Buist IA, Potter SG, Trudel BK, Shelnutt SR, Walker AH, Scholz DK, Brandvik PJ, Fritt-Rasmussen J, Allen AA, Smith P (2013) In situ burning in ice-affected waters: state of knowledge report. International Association of Oil and Gas Porducers. 209-2015 Blackfriars Road, London SE1 8NL 09:55. http://www.arcticresponsetechnology.org/. Accessed 24.11.2020

[CR15] Carls MG, Holland L, Larsen M, Collier TK, Scholz NL, Incardona JP (2008). Fish embryos are damaged by dissolved PAHs, not oil particles. Aquat Toxicol.

[CR16] Cohen A (2001). The effect of different oil spill remediation techniques on petroleum hydrocarbon elimination in Australian bass ( *Macquaria novemaculeata*). Arch Environ Contam Toxicol.

[CR17] Cohen A, Gagnon MM, Nugegoda D (2005). Alterations of metabolic enzymes in australian bass, *Macquaria novemaculeata*, after exposure to petroleum hydrocarbons. Arch Environ Contam Toxicol.

[CR18] Cohen A, Gagnon MM, Nugegoda D (2006). Oil spill remediation techniques can have different impacts on mixed function oxygenase enzyme activities in fish. Bull Environ Contam Toxicol.

[CR19] Couillard CM, Lee K, Légaré B, King TL (2005). Effect of dispersant on the composition of the water-accommodated fraction of crude oil and its toxicity to larval marine fish. Environ Toxicol Chem.

[CR20] de Soysa TY, Ulrich A, Friedrich T, Pite D, Compton SL, Ok D, Bernardos RL, Downes GB, Hsieh S, Stein R, Lagdameo MC, Halvorsen K, Kesich L-R, Barresi MJ (2012). Macondo crude oil from the Deepwater Horizon oil spill disrupts specific developmental processes during zebrafish embryogenesis. BMC Biol.

[CR21] Dupuis A, Ucan-Marin F (2015) A literature review on the aquatic toxicology of petroleum oil: An overview of oil properties and effects to aquatic biota. DFO Can. Sci. Advis. Secr. CSAS Res Doc 2015007 Vi 52 P

[CR22] Echols BS, Smith AJ, Gardinali PR, Rand GM (2015). Acute aquatic toxicity studies of Gulf of Mexico water samples collected following the Deepwater Horizon incident (May 12, 2010 to December 11, 2010). Chemosphere.

[CR23] Eganhouse RP, Calder JA (1976). The solubility of medium molecular weight aromatic hydrocarbons and the effects of hydrocarbon co-solutes and salinity. Geochim Cosmochim Acta.

[CR24] Ekperusi AO, Onyena AP, Akpudo MY, Peter CC, Akpoduado CO, Ekperusi OH (2019) In-situ burning as an oil spill control measure and its effect on the environment. Presented at the SPE Nigeria Annual International Conference and Exhibition, Society of Petroleum Engineers. 10.2118/198777-MS

[CR25] Ellman GL, Courtney KD, Andres V, Featherstone RM (1961). A new and rapid colorimetric determination of acetylcholinesterase activity. Biochem Pharmacol.

[CR26] European Union (2010). Directive 2010/63/EU of the European Parliament and of the Council of 22 September 2010 on the Protection of Animals Used for Scientific Purposes. Off J Eur Union.

[CR27] Faksness L-G, Hansen BH, Altin D, Brandvik PJ (2012). Chemical composition and acute toxicity in the water after in situ burning – a laboratory experiment. Mar Pollut Bull.

[CR28] Fingas M (2011) Introduction to oil chemistry and properties, chapter 3, handbook of oil spill science and technology. Elsevier Inc. 10.1016/B978-1-85617-943-0.10003-6

[CR29] Fritt-Rasmussen J (2010) In situ burning of Arctic marine oil spills: ignitability of various oil types weathered at different ice conditions. A combined laboratory and field study. Technical University of Denmark, Department of Civil Engineering

[CR30] Fritt-Rasmussen J, Brandvik PJ, Villumsen A, Stenby EH (2012). Comparing ignitability for in situ burning of oil spills for an asphaltenic, a waxy and a light crude oil as a function of weathering conditions under arctic conditions. Cold Reg Sci Technol.

[CR31] Fritt-Rasmussen J, Wegeberg S, Gustavson K (2015) Review on burn residues from i*n situ* burning of oil spills in relation to arctic waters. Water Air Soil Pollut 226. 10.1007/s11270-015-2593-1

[CR32] Fritt-Rasmussen J, Wegeberg S, Gustavson K, Rist Sørheim K, Jørgensen PS, Daling K, Tonteri O, Holst-Andersen JP (2018) Oil spills with HFO in cold/Arctic environment. Nordic Council of Ministers. 10.6027/6d2d9a0d-en

[CR33] Gao D, Wu M, Wang C, Wang Y, Zuo Z (2015). Chronic exposure to low benzo [a] pyrene level causes neurodegenerative disease-like syndromes in zebrafish (*Danio rerio*). Aquat Toxicol.

[CR34] Hansen BH, Farkas J, Nordtug T, Altin D, Brakstad OG (2018). Does microbial biodegradation of water-soluble components of oil reduce the toxicity to early life stages of fish?. Environ Sci Technol.

[CR35] Haverroth GMB, Welang C, Mocelin RN, Postay D, Bertoncello KT, Franscescon F, Rosemberg DB, Dal Magro J, Dalla Corte CL (2015). Copper acutely impairs behavioral function and muscle acetylcholinesterase activity in zebrafish (*Danio rerio*). Ecotoxicol Environ Saf.

[CR36] Hicken CE, Linbo TL, Baldwin DH, Willis ML, Myers MS, Holland L, Larsen M, Stekoll MS, Rice SD, Collier TK, Scholz NL, Incardona JP (2011). Sublethal exposure to crude oil during embryonic development alters cardiac morphology and reduces aerobic capacity in adult fish. Proc Natl Acad Sci.

[CR37] Hodson PV (2017). The toxicity to fish embryos of PAH in crude and refined oils. Arch Environ Contam Toxicol.

[CR38] Holmsen SH (2019) A Arctic environment, 6:59. Available online: https://ntnuopen.ntnu.no/ntnu-xmlui/. Accessed 17.07.2019.

[CR39] Hook SE, Osborn HL (2012). Comparison of toxicity and transcriptomic profiles in a diatom exposed to oil, dispersants, dispersed oil. Aquat Toxicol.

[CR40] Howe K, Clark MD, Torroja CF, Torrance J, Berthelot C, Muffato M, Collins JE, Humphray S, McLaren K, Matthews L (2013). The zebrafish reference genome sequence and its relationship to the human genome. Nature.

[CR41] Incardona JP (2017). Molecular mechanisms of crude oil developmental toxicity in fish. Arch Environ Contam Toxicol.

[CR42] Incardona JP, Collier TK, Scholz NL (2004). Defects in cardiac function precede morphological abnormalities in fish embryos exposed to polycyclic aromatic hydrocarbons. Toxicol Appl Pharmacol.

[CR43] Incardona JP, Day HL, Collier TK, Scholz NL (2006). Developmental toxicity of 4-ring polycyclic aromatic hydrocarbons in zebrafish is differentially dependent on AH receptor isoforms and hepatic cytochrome P4501A metabolism. Toxicol Appl Pharmacol.

[CR44] Incardona JP, Swarts TL, Edmunds RC, Linbo TL, Aquilina-Beck A, Sloan CA, Gardner LD, Block BA, Scholz NL (2013). Exxon Valdez to Deepwater Horizon: comparable toxicity of both crude oils to fish early life stages. Aquat Toxicol.

[CR45] Incardona JP, Gardner LD, Linbo TL, Brown TL, Esbaugh AJ, Mager EM, Stieglitz JD, French BL, Labenia JS, Laetz CA (2014). Deepwater Horizon crude oil impacts the developing hearts of large predatory pelagic fish. Proc Natl Acad Sci.

[CR101] Jee J-H, Kang J-C (2003) Effects of Phenanthrene Exposure on the Acetylcholinesterase Activity of Olive Flounder (Paralichthys olivaceus). Fisheries and Aquatic Sciences 6:225–227. 10.5657/fas.2003.6.4.225

[CR46] Jett DA, Navoa RV, Lyons MA (1999). Additive inhibitory action of chlorpyrifos and polycyclic aromatic hydrocarbons on acetylcholinesterase activity in vitro. Toxicol Lett.

[CR47] Johann S, Nüßer L, Goßen M, Hollert H, Seiler TB (2020). Differences in biomarker and behavioral responses to native and chemically dispersed crude and refined fossil oils in zebrafish early life stages. Sci Total Environ.

[CR48] Jørgensen KS, Kreutzer A, Lehtonen KK, Kankaanpää H, Rytkönen J, Wegeberg S, Gustavson K, Fritt-Rasmussen J, Truu J, Kõuts T (2019). The EU Horizon 2020 project GRACE: integrated oil spill response actions and environmental effects. Environ Sci Eur.

[CR49] Jung J-H, Hicken CE, Boyd D, Anulacion BF, Carls MG, Shim WJ, Incardona JP (2013). Geologically distinct crude oils cause a common cardiotoxicity syndrome in developing zebrafish. Chemosphere.

[CR50] Kais B, Stengel D, Batel A, Braunbeck T (2015). Acetylcholinesterase in zebrafish embryos as a tool to identify neurotoxic effects in sediments. Environ Sci Pollut Res.

[CR51] Kennedy CJ, Farrell AP (2006). Effects of exposure to the water-soluble fraction of crude oil on the swimming performance and the metabolic and ionic recovery postexercise in pacific herring (*Clupea pallasi*). Environ Toxicol Chem.

[CR52] Khursigara AJ, Perrichon P, Bautista NM, Burggren WW, Esbaugh AJ (2017). Cardiac function and survival are affected by crude oil in larval red drum, Sciaenops ocellatus. Sci Total Environ.

[CR53] King TL, Robinson B, Cui F, Boufadel M, Lee K, Clyburne JAC (2017). An oil spill decision matrix in response to surface spills of various bitumen blends. Environ Sci Process Impacts.

[CR54] Kristensen M, Johnsen AR, Christensen JH (2015). Marine biodegradation of crude oil in temperate and Arctic water samples. J Hazard Mater.

[CR55] Le Bihanic F, Clérandeau C, Le Menach K, Morin B, Budzinski H, Cousin X, Cachot J (2014). Developmental toxicity of PAH mixtures in fish early life stages. Part II: adverse effects in Japanese medaka. Environ Sci Pollut Res.

[CR56] Lee LS, Rao PSC, Okuda I (1992). Equilibrium partitioning of polycyclic aromatic hydrocarbons from coal tar into water. Environ Sci Technol.

[CR57] Lee K, Nedwed T, Prince RC (2011) Lab tests on the biodegradation rates of chemically dispersed oil must consider natural dilution. International Oil Spill Conference 2011:abs245. 10.7901/2169-3358-2011-1-245

[CR58] Legradi J, El Abdellaoui N, Van Pomeren M, Legler J (2015). Comparability of behavioural assays using zebrafish larvae to assess neurotoxicity. Environ Sci Pollut Res.

[CR59] Legradi JB, Di Paolo C, Kraak MHS, Van Der Geest HG, Schymanski EL, Williams AJ, Dingemans MML, Massei R, Brack W, Cousin X (2018). An ecotoxicological view on neurotoxicity assessment. Environ Sci Eur.

[CR60] Mager EM, Esbaugh AJ, Stieglitz JD, Hoenig R, Bodinier C, Incardona JP, Scholz NL, Benetti DD, Grosell M (2014). Acute embryonic or juvenile exposure to Deepwater Horizon crude oil impairs the swimming performance of Mahi-Mahi (*Coryphaena hippurus*). Environ Sci Technol.

[CR61] Martin JD, Adams J, Hollebone B, King T, Brown RS, Hodson PV (2014). Chronic toxicity of heavy fuel oils to fish embryos using multiple exposure scenarios. Environ Toxicol Chem.

[CR62] Meyer-Alert H, Ladermann K, Larsson M, Schiwy S, Hollert H, Keiter SH (2018). A temporal high-resolution investigation of the Ah-receptor pathway during early development of zebrafish (*Danio rerio*). Aquat Toxicol.

[CR63] Nahrgang J, Dubourg P, Frantzen M, Storch D, Dahlke F, Meador JP (2016). Early life stages of an arctic keystone species (Boreogadus saida) show high sensitivity to a water-soluble fraction of crude oil. Environ Pollut.

[CR64] OECD (2013) Test No. 236: Fish Embryo Acute Toxicity (FET) Test

[CR65] Oliveira M, Pacheco M, Santos MA (2007) Cytochrome P4501A, genotoxic and stress responses in golden grey mullet (*Liza aurata*) following short-term exposure to phenanthrene. Chemosphere 66:1284–1291. 10.1016/j.chemosphere.2006.07.024 10.1016/j.chemosphere.2006.07.024 16930669

[CR66] Pauka LM, Maceno M, Rossi SC, Silva de Assis HC (2011). Embryotoxicity and biotransformation responses in zebrafish exposed to water-soluble fraction of crude oil. Bull Environ Contam Toxicol.

[CR67] Perrichon P, Le Bihanic F, Bustamante P, Le Menach K, Budzinski H, Cachot J, Cousin X (2014) Influence of sediment composition on PAH toxicity using zebrafish (*Danio rerio*) and Japanese medaka (*Oryzias latipes*) embryo-larval assays. Environ Sci Pollut Res 21:13703–13719. 10.1007/s11356-014-3502-7 10.1007/s11356-014-3502-7 25175355

[CR68] Perrichon P, Le Menach K, Akcha F, Cachot J, Budzinski H, Bustamante P (2016). Toxicity assessment of water-accommodated fractions from two different oils using a zebrafish (*Danio rerio*) embryo-larval bioassay with a multilevel approach. Sci Total Environ.

[CR69] Philibert DA, Philibert CP, Lewis C, Tierney KB (2016). Comparison of diluted bitumen (Dilbit) and conventional crude oil toxicity to developing zebrafish. Environ Sci Technol.

[CR70] Potter DW, Pawliszyn J (1994). Rapid determination of polyaromatic hydrocarbons and polychlorinated biphenyls in water using solid-phase microextraction and GC/MS. Environ Sci Technol.

[CR71] Prince RC (2015). Oil spill dispersants: boon or bane?. Environ Sci Technol.

[CR72] Ramachandran SD, Hodson PV, Khan CW, Lee K (2004). Oil dispersant increases PAH uptake by fish exposed to crude oil. Ecotoxicol Environ Saf.

[CR73] Redman AD, Parkerton TF (2015). Guidance for improving comparability and relevance of oil toxicity tests. Mar Pollut Bull.

[CR74] Redman AD, Butler JD, Letinski DJ, Di Toro DM, Paumen ML, Parkerton TF (2018). Technical basis for using passive sampling as a biomimetic extraction procedure to assess bioavailability and predict toxicity of petroleum substances. Chemosphere.

[CR75] Sanni S, Björkblom C, Jonsson H, Godal BF, Liewenborg B, Lyng E, Pampanin DM (2017). I: biomarker quantification in fish exposed to crude oil as input to species sensitivity distributions and threshold values for environmental monitoring. Mar Environ Res.

[CR76] Saranjampour P, Vebrosky EN, Armbrust KL (2017). Salinity impacts on water solubility andn-octanol/water partition coefficients of selected pesticides and oil constituents. Environ Toxicol Chem.

[CR77] Schiwy S, Bräunig J, Alert H, Hollert H, Keiter SH (2014). A novel contact assay for testing aryl hydrocarbon receptor (AhR)-mediated toxicity of chemicals and whole sediments in zebrafish (Danio rerio) embryos. Environ Sci Pollut Res.

[CR78] Scholz S, Fischer S, Gündel U, Küster E, Luckenbach T, Voelker D (2008). The zebrafish embryo model in environmental risk assessment—applications beyond acute toxicity testing. Environ Sci Pollut Res.

[CR79] Scott JA, Incardona JP, Pelkki K, Shepardson S, Hodson PV (2011) AhR2-mediated, CYP1A-independent cardiovascular toxicity in zebrafish (*Danio rerio*) embryos exposed to retene. Aquat Toxicol 101:165–174. 10.1016/j.aquatox.2010.09.016 10.1016/j.aquatox.2010.09.016 21040984

[CR80] SDS sheet (2013) Finasol OSR 52. Safety data sheet according to regulation (EC) No 1970/2006. SDS # 30034

[CR81] Seiler T-B, Best N, Fernqvist MM, Hercht H, Smith KEC, Braunbeck T, Mayer P, Hollert H (2014). PAH toxicity at aqueous solubility in the fish embryo test with *Danio rerio* using passive dosing. Chemosphere.

[CR82] Singer MM, Aurand D, Bragin GE, Clark JR, Coelho GM, Sowby ML, Tjeerdema RS (2000). Standardization of the preparation and quantitation of water-accommodated fractions of petroleum for toxicity testing. Mar Pollut Bull.

[CR83] Sørensen L, Hansen BH, Farkas J, Donald C, Robson W, Tonkin A, Meier S, Rowland SJ (2019) Accumulation and toxicity of monoaromatic petroleum hydrocarbons in early life stages of cod and haddock. Environemntal Pollution. 10.1016/j.envpol.2019.04.126 10.1016/j.envpol.2019.04.126 31078960

[CR84] Soreq H, Seidman S (2001). Acetylcholinesterase — new roles for an old actor. Nat Rev Neurosci.

[CR85] Spaulding ML (2017). State of the art review and future directions in oil spill modeling. Mar Pollut Bull.

[CR86] Stieglitz JD, Mager EM, Hoenig RH, Alloy M, Esbaugh AJ, Bodinier C, Benetti DD, Roberts AP, Grosell M (2016). A novel system for embryo-larval toxicity testing of pelagic fish: applications for impact assessment of Deepwater Horizon crude oil. Chemosphere.

[CR87] Strähle U, Scholz S, Geisler R, Greiner P, Hollert H, Rastegar S, Schumacher A, Selderslaghs I, Weiss C, Witters H, Braunbeck T (2012). Zebrafish embryos as an alternative to animal experiments—a commentary on the definition of the onset of protected life stages in animal welfare regulations. Reprod Toxicol.

[CR88] Tairova Z, Frantzen M, Mosbech A, Arukwe A, Gustavson K (2019) Effects of water accommodated fraction of physically and chemically dispersed heavy fuel oil on beach spawning capelin (*Mallotus villosus*). Mar Environ Res 147:62–71. 10.1016/j.marenvres.2019.03.010 10.1016/j.marenvres.2019.03.010 31047709

[CR89] Tang Y, Donnelly KC, Tiffany-Castiglioni E, Mumtaz MM (2003). Neurotoxicity of polycyclic aromatic hydrocarbons and simple chemical mixtures. J Toxic Environ Health A.

[CR90] Tilton FA, Bammler TK, Gallagher EP (2011). Swimming impairment and acetylcholinesterase inhibition in zebrafish exposed to copper or chlorpyrifos separately, or as mixtures. Comp Biochem Physiol C Toxicol Pharmacol.

[CR91] van der Oost R, Beyer J, Vermeulen NPE (2003). Fish bioaccumulation and biomarkers in environmental risk assessment: a review. Environ Toxicol Pharmacol.

[CR92] Velki M, Meyer-Alert H, Seiler T-B, Hollert H (2017). Enzymatic activity and gene expression changes in zebrafish embryos and larvae exposed to pesticides diazinon and diuron. Aquat Toxicol.

[CR93] Vignet C, Trenkel VM, Vouillarmet A, Bricca G, Bégout M-L, Cousin X (2017). Changes in brain monoamines underlie behavioural disruptions after zebrafish diet exposure to polycyclic aromatic hydrocarbons environmental mixtures. Int J Mol Sci.

[CR94] Wegeberg S, Fritt-Rasmussen J, Boertmann D (2017) Oil spill response in Greenland: net environmental benefit analysis, neba, and environmental monitoring scientific report from DCE–Danish Centre for Environment and Energy No. 221

[CR95] Wegeberg S, Fritt-Rasmussen J, Gustavson K (2018a) Report on results from field experiments in Greenland. Deliverable 4.14. Prepared under contract from the European Commission Contract n° 679266, Research and Innovation Action Innovation and Networks Executive Agency, Horizon 2020 BG-2014-2015/BG2015-2; https://www.grace-oil-project.eu/en-US/About/Deliverables

[CR96] Wegeberg S, Johnson A, Aamand J, Lassen PJ, Gosewinkel U, Fritt-Rasmussen J, Riget F, Gustavson K, Mosbech A (2018b) Arctic marine potential of microbial oil degradation Aarhus University, DCE – Danish Centre for Environment and Energy, 54 pp. Scientific Report from DCE – Danish Centre for Environment and Energy No. 271

[CR97] Whitehouse BG (1984). The effects of temperature and salinity on the aqueous solubility of polynuclear aromatic hydrocarbons. Mar Chem.

[CR98] Xu EG, Mager EM, Grosell M, Pasparakis C, Schlenker LS, Stieglitz JD, Benetti D, Hazard ES, Courtney SM, Diamante G, Freitas J, Hardiman G, Schlenk D (2016). Time- and oil-dependent transcriptomic and physiological responses to Deepwater Horizon oil in Mahi-Mahi (*Coryphaena hippurus*) Embryos and Larvae. Environ Sci Technol.

[CR99] Xu EG, Khursigara AJ, Magnuson J, Hazard ES, Hardiman G, Esbaugh AJ, Roberts AP, Schlenk D (2017). Larval red drum (*Sciaenops ocellatus*) sublethal exposure to weathered Deepwater Horizon crude oil: developmental and transcriptomic consequences. Environ Sci Technol.

[CR100] Yen J, Donerly S, Levin ED, Linney EA (2011). Differential acetylcholinesterase inhibition of chlorpyrifos, diazinon and parathion in larval zebrafish. Neurotoxicol Teratol.

