# Supplementary material for: Comparative toxicity assessment of in situ burn residues to initial and dispersed heavy fuel oil using zebrafish embryos as test organisms
Source: Environ Sci Pollut Res Int. 2020 Dec 3;28(13):16198–213. doi: 10.1007/s11356-020-11729-5 (PMC7969557; doi:10.1007/s11356-020-11729-5)
Supplement: Supplementary file 1 — (DOCX 234 kb). [file 11356_2020_11729_MOESM1_ESM.docx]

**Supplementary information**

**Comparative toxicity assessment of in situ burn residues to initial and dispersed heavy fuel oil using zebrafish embryos as test organisms**

Sarah Johann^a,e^, Mira Goßen^a,e^, Leonie Nüßer^a^, Valentina Selja^b^, Kim Gustavson^c^, Janne Fritt-Rasmussen^c^, Susse Wegeberg^c^, Tomasz Maciej Ciesielski^d^, Bjørn Munro Jenssen^d^, Henner Hollert^e^, and Thomas-Benjamin Seiler^a^

^a^Department of Ecosystem Analysis, Institute for Environmental Research, RWTH Aachen University, Worringerweg 1, 52074 Aachen, Germany

^b^Department of Biology, Josip Juraj Strossmayer University of Osijek, Cara Hadrijana 8/A, 31000 Osijek, Croatia

^c^Department of Bioscience, Aarhus University, Frederiksborgvej 399, 4000 Roskilde, Denmark

^d^Department of Biology, Norwegian University of Science and Technology, 7491 Trondheim, Norway

^e^Department of Evolutionary Ecology and Environmental Toxicology, Goethe University Frankfurt, Max-von-Laue-Str. 13, 60438 Frankfurt am Main, Germany

corresponding authors: Sarah Johann & Thomas-Benjamin Seiler

email addresses of corresponding authors: sarah.johann@rwth-aachen.de & seiler@bio5.rwth-aachen.de

**Jounal: Environmental Science and Pollution research**

## 1. Acute fish embryo toxicity

##

**Figure S1 Acute toxicity in zebrafish larvae (120 hpf) exposed to water-accommodated fraction of initial, burned and dispersed IFO180.** Dots and error denote mean and SD of effects (lethal + sublethal) of 2 (burn residue) - 3 independent replicates. Triangles show the negative (artificial medium, pointing downwards) and positive controls (3,4-dichloraniline). Sigmoidal concentration-response curves were added in GraphPad Prism 6 using the 4-parameter non-linear regression model, top and bottom variables were set to 100 and 0, respectively. Dotted lines indicate 95 % confidence band (Equation: Y= 1/(1+10^((LogEC50-X)*HillSlope).

Table S1 Morphological sublethal and lethal effects zebrafish embryos exposed to a dilution series of initial WAF of IFO180 over time. Data represent mean and standard deviation (n=3) of % effects observed during acute fish embryo toxicity test.

|  |  | **IFO 180 LEWAF [% effects]** | | | | |  | |
| --- | --- | --- | --- | --- | --- | --- | --- | --- |
|  | *exposure dilution [%] of stock* | *66.7* | *50.0* | *33.3* | *25.0* | *16.7* |  |  |
|  |  |  |  |  |  |  |  |  |
| **48 hpf (*n=2)** | edema (heart) | 18.3 ± 31.8 | 8.3 ± 14.4 |  |  |  |  |  |
|  | edema (yolk sac) | 51.7 ± 7.6 | 26.7 ± 18.9 | 6.7 ± 7.6 |  |  |  |  |
|  | slow heartbeat |  |  |  |  |  |  |  |
|  | heart deformation |  |  |  |  |  |  |  |
|  | slow blood flow | 3.3 ± 5.8 | 5.0 ± 0.0 |  |  |  |  |  |
|  | blood congestion | 8.3 ± 7.6 | 8.3 ± 5.8 | 6.7 ± 7.6 |  | 1.7 ± 2.9 |  |  |
|  | spine deformation | 1.7 ± 2.9 |  |  |  |  |  |  |
|  | no blood flow |  |  |  |  |  |  |  |
|  | no heartbeat |  |  |  |  |  |  |  |
|  | coagulation | 3.3 ± 2.9 | 1.7 ± 2.9 | 1.7 ± 2.9 | 5.0 ± 5.0 | 1.7 ± 2.9 |  |  |
|  |  |  |  |  |  |  |  |  |
| **72 hpf** | edema (heart) | 70.0 ± 26.5 | 38.3 ± 18.9 | 20.0 ± 8.7 | 5.0 ± 8.7 |  |  |  |
|  | edema (yolk sac) | 56.7 ± 25.6 | 43.3 ± 27.5 | 16.7 ± 15.3 | 3.3 ± 5.8 |  |  |  |
|  | slow heartbeat |  | 1.7 ± 2.9 |  |  |  |  |  |
|  | heart deformation | 11.7 ± 10.4 |  | 3.3 ± 5.8 | 1.7 ± 2.9 |  |  |  |
|  | slow blood flow | 3.3 ± 5.8 |  |  |  |  |  |  |
|  | blood congestion | 28.3 ± 15.3 | 25.0 ± 5.0 | 11.7 ± 12.6 | 10.0 ± 0.0 |  |  |  |
|  | spine deformation |  |  |  |  |  |  |  |
|  | no blood flow | 10.0 ± 8.7 |  | 1.7 ± 2.9 |  |  |  |  |
|  | no heartbeat |  |  | 1.7 ± 2.9 |  |  |  |  |
|  | coagulation | 3.3 ± 2.9 | 1.7 ± 2.9 | 3.3 ± 5.8 | 5.0 ± 5.0 | 1.7 ± 2.9 |  |  |
|  |  |  |  |  |  |  |  |  |
| **96 hpf** | edema (heart) | 88.3 ± 11.5 | 83.3 ± 14.4 | 41.7 ± 35.5 | 11.7 ± 12.6 |  |  |  |
|  | edema (yolk sac) | 15.0 ± 10.0 | 11.7 ± 7.6 |  |  |  |  |  |
|  | slow heartbeat | 5.0 ± 5.0 | 8.3 ± 7.6 | 1.7 ± 2.9 |  |  |  |  |
|  | heart deformation | 46.7 ± 7.6 | 30.0 ± 20.0 | 11.7 ± 10.4 | 1.7 ± 2.9 |  |  |  |
|  | slow blood flow |  |  | 1.7 ± 2.9 |  |  |  |  |
|  | blood congestion | 11.7 ± 5.8 | 16.7 ± 7.6 | 8.3 ± 10.4 | 1.7 ± 2.9 |  |  |  |
|  | spine deformation | 13.3 ± 2.9 | 13.3 ± 10.4 | 5.0 ± 8.7 |  |  |  |  |
|  | no blood flow | 61.7 ± 20.2 | 41.7 ± 18.9 | 13.3 ± 5.8 | 5.0 ± 5.0 |  |  |  |
|  | no heartbeat | 15.0 ± 18.0 |  | 3.3 ± 2.9 |  |  |  |  |
|  | coagulation | 10.0 ± 13.2 | 1.7 ± 2.9 | 5.0 ± 5.0 | 8.3 ± 10.4 | 3.3 ± 2.9 |  |  |
|  |  |  |  |  |  |  |  |  |
| **120 hpf** | edema (heart) | 28.3 ± 10.4 | 45.0 ± 27.8 | 46.7 ± 18.9 | 15.0 ± 13.2 | 6.7 ± 11.5 |  |  |
|  | edema (yolk sac) | 11.7 ± 16.1 | 1.7 ± 2.9 |  | 1.7 ± 2.9 |  |  |  |
|  | slow heartbeat | 6.7 ± 7.6 | 5.0 ± 8.7 | 11.7 ± 12.6 | 8.3 ± 5.8 | 3.3 ± 2.9 |  |  |
|  | heart deformation | 10.0 ± 5.0 | 25.0 ± 25.0 | 18.3 ± 18.9 | 11.7 ± 5.8 | 1.7 ± 2.9 |  |  |
|  | slow blood flow |  |  | 1.7 ± 2.9 |  |  |  |  |
|  | blood congestion | 1.7 ± 2.9 | 1.7 ± 2.9 | 5.0 ± 8.7 | 16.7 ± 24.7 |  |  |  |
|  | spine deformation | 21.7 ± 15.3 | 28.3 ± 27.5 | 31.7 ± 32.5 | 6.7 ± 11.5 | 6.7 ± 7.6 |  |  |
|  | no blood flow | 30.0 ± 18.0 | 31.7 ± 17.6 | 36.7 ± 47.3 | 20.0 ± 15.0 | 6.7 ± 7.6 |  |  |
|  | no heartbeat | 21.7 ± 12.6 | 15.0 ± 10.0 | 16.7 ± 20.8 | 6.7 ± 11.5 | 3.3 ± 5.8 |  |  |
|  | coagulation | 68.3 ± 16.1 | 51.7 ± 29.3 | 25.0 ± 34.6 | 11.7 ± 16.1 | 3.3 ± 2.9 |  |  |

Table S1 continued

|  |  | **burned IFO 180 LEWAF [% effects]** | | | | |  | |
| --- | --- | --- | --- | --- | --- | --- | --- | --- |
|  | *exposure dilution [%] of stock* | *100.0* | *66.7* | *50.0* | *33.3* | *25.0* |  |  |
|  |  |  |  |  |  |  |  |  |
| **48 hpf** | edema (heart) | 6.7 ± 11.5 | 8.3 ± 14.4 | 1.7 ± 2.9 | 5.0 ± 8.7 | 6.7 ± 11.5 |  |  |
|  | edema (yolk sac) | 5.0 ± 5.0 | 8.3 ± 7.6 | 5.0 ± 8.7 | 1.7 ± 2.9 | 8.3 ± 7.6 |  |  |
|  | slow heartbeat | 1.7 ± 2.9 |  |  |  |  |  |  |
|  | heart deformation | 5.0 ± 8.7 | 3.3 ± 5.8 | 1.7 ± 2.9 |  |  |  |  |
|  | slow blood flow |  |  |  |  |  |  |  |
|  | blood congestion |  |  |  |  |  |  |  |
|  | spine deformation |  |  |  |  |  |  |  |
|  | no blood flow |  |  |  |  |  |  |  |
|  | no heartbeat |  |  |  |  |  |  |  |
|  | coagulation |  | 3.3 ± 5.8 | 6.7 ± 7.6 |  | 5.0 ± 5.0 |  |  |
|  |  |  |  |  |  |  |  |  |
| **72 hpf** | edema (heart) | 33.3 ± 12.6 | 18.3 ± 20.2 | 13.3 ± 23.1 | 21.8 ± 33.2 | 8.3 ± 14.4 |  |  |
|  | edema (yolk sac) | 11.7 ± 11.5 |  | 1.7 ± 2.9 |  |  |  |  |
|  | slow heartbeat |  |  |  |  |  |  |  |
|  | heart deformation | 11.7 ± 5.8 | 6.7 ± 11.5 |  |  |  |  |  |
|  | slow blood flow | 10.0 ± 8.7 | 6.7 ± 2.9 |  |  |  |  |  |
|  | blood congestion | 3.3 ± 5.8 | 3.3 ± 2.9 |  |  |  |  |  |
|  | spine deformation | 1.7 ± 2.9 |  |  |  |  |  |  |
|  | no blood flow | 8.3 ± 10.4 |  |  |  |  |  |  |
|  | no heartbeat |  |  |  |  |  |  |  |
|  | coagulation |  | 3.3 ± 5.8 | 6.7 ± 7.6 |  | 5.0 ± 5.0 |  |  |
|  |  |  |  |  |  |  |  |  |
| **96 hpf** | edema (heart) | 58.3 ± 17.6 | 31.7 ± 27.5 | 45.0 ± 32.8 | 28.3 ± 49.1 | 10.0 ± 17.3 |  |  |
|  | edema (yolk sac) | 1.7 ± 2.9 |  |  |  |  |  |  |
|  | slow heartbeat | 11.7 ± 10.4 | 5.0 ± 5.0 | 5.0 ± 5.0 | 1.7 ± 2.9 |  |  |  |
|  | heart deformation | 28.3 ± 7.6 | 3.3 ± 2.9 | 3.3 ± 5.8 |  |  |  |  |
|  | slow blood flow | 3.3 ± 5.8 |  | 3.3 ± 5.8 |  |  |  |  |
|  | blood congestion |  | 1.7 ± 2.9 |  |  |  |  |  |
|  | spine deformation |  |  |  |  |  |  |  |
|  | no blood flow | 26.7 ± 15.3 | 6.7 ± 5.8 | 5.0 ± 5.0 | 3.3 ± 5.8 | 1.7 ± 2.9 |  |  |
|  | no heartbeat | 11.7 ± 10.4 | 1.7 ± 2.9 | 1.7 ± 2.9 | 1.7 ± 2.9 | 1.7 ± 2.9 |  |  |
|  | coagulation | 5.0 ± 8.7 | 3.3 ± 5.8 | 6.7 ± 7.6 |  | 5.0 ± 5.0 |  |  |
|  |  |  |  |  |  |  |  |  |
| **120 hpf** | edema (heart) | 57.5 ± 24.7 | 52.5 ± 3.5 | 32.5 ± 38.9 | 20.0 ± 7.1 | 2.5 ± 3.5 |  |  |
|  | edema (yolk sac) | 27.5 ± 3.5 | 2.5 ± 3.5 |  | 12.5 ± 10.6 |  |  |  |
|  | slow heartbeat | 7.5 ± 10.6 | 10.0 ± 7.1 | 12.5 ± 17.7 | 5.0 ± 0.0 | 2.5 ± 3.5 |  |  |
|  | heart deformation | 22.5 ± 10.6 | 15.0 ± 14.1 | 20.0 ± 28.3 | 7.5 ± 10.6 |  |  |  |
|  | slow blood flow |  |  | 2.5 ± 3.5 |  |  |  |  |
|  | blood congestion |  |  |  | 2.5 ± 3.5 |  |  |  |
|  | spine deformation | 17.5 ± 24.7 | 30.0 ± 42.4 | 32.5 ± 46.0 | 2.5 ± 3.5 | 5.0 ± 0.0 |  |  |
|  | no blood flow | 57.5 ± 10.6 | 35.0 ± 21.2 | 45.0 ± 63.6 | 20.0 ± 0.0 | 2.5 ± 3.5 |  |  |
|  | no heartbeat | 30.0 ± 7.1 | 20.0 ± 7.1 | 20.0 ± 28.3 | 5 ± 7.1 |  |  |  |
|  | coagulation | 35.0 ± 21.2 | 25.0 ± 21.2 | 20.0 ± 21.2 | 7.5 ± 10.6 | 20.0 ± 21.2 |  |  |

Table S1 continued

|  |  | **IFO 180 CEWAF [% effects]** | | | | |  | | | |  | | |  |
| --- | --- | --- | --- | --- | --- | --- | --- | --- | --- | --- | --- | --- | --- | --- |
|  | *exposure dilution [%] of stock* | *12.5* | *6.3* | *4.2* | *3.1* | *1.6* | *0.8* |  |  |  |  |  |  |  |
|  |  |  |  |  |  |  |  |  |  |  |  |  |  |  |
| **48 hpf** | edema (heart) |  |  |  |  |  |  |  |  |  |  |  |  |  |
|  | edema (yolk sac) | 1.7 ± 2.9 |  |  | 1.7 ± 2.9 |  |  |  |  |  |  |  |  |  |
|  | slow heartbeat |  |  |  |  |  |  |  |  |  |  |  |  |  |
|  | heart deformation |  |  |  |  |  |  |  |  |  |  |  |  |  |
|  | slow blood flow |  |  |  |  |  |  |  |  |  |  |  |  |  |
|  | blood congestion | 1.7 ± 2.9 |  |  | 1.7 ± 2.9 |  |  |  |  |  |  |  |  |  |
|  | spine deformation |  |  |  |  |  |  |  |  |  |  |  |  |  |
|  | no blood flow |  |  |  |  |  |  |  |  |  |  |  |  |  |
|  | no heartbeat |  |  |  |  |  |  |  |  |  |  |  |  |  |
|  | coagulation | 3.3 ± 2.9 | 1.7 ± 2.9 | 5.0 ± 5.0 |  |  | 2.5 ± 3.5 |  |  |  |  |  |  |  |
|  |  |  |  |  |  |  |  |  |  |  |  |  |  |  |
| **72 hpf** | edema (heart) |  |  |  |  |  |  |  |  |  |  |  |  |  |
|  | edema (yolk sac) | 31.7 ± 54.8 | 21.7 ± 37.5 | 3.3 ± 5.8 | 5.0 ± 5.0 |  |  |  |  |  |  |  |  |  |
|  | slow heartbeat |  |  |  |  |  |  |  |  |  |  |  |  |  |
|  | heart deformation | 93.3 ± 2.9 | 55.0 ± 32.8 | 31.7 ± 7.6 | 16.7 ± 17.6 |  |  |  |  |  |  |  |  |  |
|  | slow blood flow | 20.0 ± 18.0 | 3.3 ± 5.8 |  |  |  |  |  |  |  |  |  |  |  |
|  | blood congestion | 1.7 ± 2.9 |  |  |  |  |  |  |  |  |  |  |  |  |
|  | spine deformation | 10.0 ± 17.3 |  | 1.7 ± 2.9 |  |  |  |  |  |  |  |  |  |  |
|  | no blood flow | 21.7 ± 20.8 |  |  |  |  |  |  |  |  |  |  |  |  |
|  | no heartbeat | 1.7 ± 2.9 |  |  | 1.7 ± 2.9 |  |  |  |  |  |  |  |  |  |
|  | coagulation | 1.7 ± 2.9 | 3.3 ± 2.9 | 5.0 ± 5.0 |  |  | 2.5 ± 3.5 |  |  |  |  |  |  |  |
|  |  |  |  |  |  |  |  |  |  |  |  |  |  |  |
| **96 hpf** | edema (heart) |  |  |  |  |  |  |  |  |  |  |  |  |  |
|  | edema (yolk sac) | 35.0 ± 39.7 | 12.5 ± 17.7 |  |  |  |  |  | | | |  |  |  |
|  | slow heartbeat | 3.3 ± 2.9 |  |  |  |  |  |  | | | |  |  |  |
|  | heart deformation | 53.3 ± 35.1 | 62.5 ± 3.5 | 20.0 ± 7.1 | 15.0 ± 0.0 | 2.5 ± 3.5 | 2.5 ± 3.5 |  | | | |  |  |  |
|  | slow blood flow | 5.0 ± 8.7 | 42.5 ± 24.7 | 5.0 ± 7.1 |  |  |  |  | | | |  |  |  |
|  | blood congestion |  |  |  |  |  |  |  | | | |  |  |  |
|  | spine deformation | 56.7 ± 37.5 | 15.0 ± 21.2 | 2.5 ± 3.5 |  | 2.5 ± 3.5 |  |  | | | |  |  |  |
|  | no blood flow | 48.3 ± 27.5 | 7.5 ± 10.6 |  |  |  |  |  | | | |  |  |  |
|  | no heartbeat | 5.0 ± 5.0 | 2.5 ± 3.5 |  |  |  |  |  | | | |  |  |  |
|  | coagulation | 41.7 ± 37.5 | 5.0 ± 0.0 | 7.5 ± 3.5 | 2.5 ± 3.5 |  | 7.5 ± 10.6 |  | | | |  |  |  |
|  |  |  |  |  |  |  |  |  | | | |  |  |  |
| **120 hpf** | edema (heart) |  |  |  |  |  |  |  | | | |  |  |  |
|  | edema (yolk sac) |  | 10.0 ± 13.2 | 50.0 ± 50.0 | 10.0 ± 17.3 | 1.7 ± 2.9 |  |  | | | |  |  |  |
|  | slow heartbeat |  | 13.3 ± 15.3 | 35.0 ± 26.5 |  |  |  |  | | | |  |  |  |
|  | heart deformation |  | 13.3 ± 15.3 | 75.0 ± 25.0 | 45.0 ± 36.1 | 6.7 ± 7.6 |  |  | | | |  |  |  |
|  | slow blood flow |  |  | 5.0 ± 5.0 | 3.3 ± 5.8 |  |  |  | | | |  |  |  |
|  | blood congestion |  |  | 1.7 ± 2.9 | 1.7 ± 2.9 |  |  |  | | | |  |  |  |
|  | spine deformation |  | 20.0 ± 8.7 | 58.3 ± 5.8 | 33.3 ± 38.2 | 3.3 ± 2.9 |  |  | | | |  |  |  |
|  | no blood flow |  | 11.7 ± 16.1 | 46.7 ± 27.5 | 31.7 ± 35.5 |  |  |  | | | |  |  |  |
|  | no heartbeat |  | 11.7 ± 2.9 | 8.3 ± 14.4 | 1.7 ± 2.9 | 1.7 ± 2.9 |  |  | | | |  |  |  |
|  | coagulation | 100 ± 0.0 | 60.0 ± 25.0 | 6.7 ± 5.8 | 5.0 ± 5.0 |  | 7.5 ± 10.6 |  | | | |  |  |  |

## 3. Larval swimming behavior alterations

**Figure S2 Alteration of zebrafish larvae (96 hpf) swimming behavior exposed to WAF dilutions of initial, burned and dispersed IFO180 in a light/dark transition test.** Independent experiments (3-4) are shown as individual lines with each line denoting the mean distance moved of 16 to 20 individual untreated (black) and WAF exposed (blue) larvae.

**Figure S3 Comparison of zebrafish larvae (96hpf) swimming alterations exposed to identical WAF dilutions of initial and burned IFO180 in a light/dark transition test.** Bars represent the mean distance moved of 16-20 larvae per experiment further averaged over 3 (initial) to 4 (burned) independent experiments. After verifying normal distribution and variance homogeneity no statistically significant differences between treatment groups were found by One-way ANOVA with Dunnett’s post-hoc test (p<0.05).

**Figure S4 Comparison of biomarker activity in zebrafish larvae exposed to identical initial and burned IFO180 WAF dilutions.** Specific 7-Ethoxyresorufin-O-deethylase (EROD) and acetylcholinesterase (AChE) activities of treatment groups were normalized to unexposed negative control (NC). Bars and error bars represent mean and standard deviation of normalized activity of 3 independent experiments. After verifying normal distribution and equal variance no statistically significant difference between treatment groups was detected using One-Way ANOVA with Dunnett’s post hoc test (p<0.05).

## 4. Chemical profile of IFO 180

### 4.1 Material and Methods

The samples of IFO180 fresh oil were analyzed for SVOC (decalins, PAHs and phenols) using GC/MS, for TPH using GC/FID, and for volatile organic compounds (VOC, C_5_–C_9_), including BTEX (benzene, toluene, ethylbenzene and xylenes), by use of P&T GC/MS (Purge and Trap Gas Chromatography Mass Spectrometry) according to a method described in Faksness et al. (2015). The GC/FID analyses were performed according to a modified EPA Method 8100 (US EPA, 1986). The semi-volatiles were quantified by modifications of EPA Method 8270D (US EPA, 2007). The mass spectrometer was operated in the selective ion monitoring mode to achieve optimum sensitivity and specificity. The quantification of target compounds was performed by internal standards addition, using average response factors (RF) for the parent compounds. The PAH and phenol alkyl homologues were quantified using the straight baseline integration of each level of alkylation and the RF for the respective parent PAH compound. The response factors were generated for all targets and surrogates versus fluorene-d_10_. A total of 35 target volatile organic compounds (VOC) in the C_5_–C_10_ range were determined by Purge and Trap (P&T) GC/MS using a modified EPA method 8260C (US EPA, 2006). The samples were spiked with SIS (toluene-d_8_ and ethylbenzene-d_8_) and RIS (chlorobenzene-d_5_). The quantification of individual compounds was performed by using the RFs of the individual compounds relative to the internal standards. All standards and samples were analyzed in a full scan mode.

### 4.2 Results

Table S2 Concentrations of individual oil compounds detected in the in the fresh oil IFO 180. The sample was analysed in duplicate and mean values are presented. TEM: total extractable material.

| Group | Compounds | IFO 180  [g kg^-1^] |
| --- | --- | --- |
| SVOC | Decalin | 0.612 |
|  | C1-decalins | 0.793 |
|  | C2-decalins | 0.668 |
|  | C3-decalins | 0.572 |
|  | C4-decalins | 0.480 |
|  | Benzo(b)thiophene | 0.006 |
| Naphtalenes | Naphthalene | 0.517 |
|  | C1-naphthalenes | 2.073 |
|  | C2-naphthalenes | 3.876 |
|  | C3-naphthalenes | 3.127 |
|  | C4-naphthalenes | 1.778 |
| 2-3 ring PAHs | Biphenyl | 0.117 |
|  | Acenaphthylene | 0.029 |
|  | Acenaphthene | 0.088 |
|  | Dibenzofuran | 0.058 |
|  | Fluorene | 0.143 |
|  | C1-fluorenes | 0.334 |
|  | C2-fluorenes | 0.580 |
|  | C3-fluorenes | 0.526 |
|  | Phenanthrene | 0.514 |
|  | Anthracene | 0.078 |
|  | C1-phenanthrenes/anthracenes | 1.376 |
|  | C2-phenanthrenes/anthracenes | 1.496 |
|  | C3-phenanthrenes/anthracenes | 0.850 |
|  | C4-phenanthrenes/anthracenes | 0.480 |
|  | Dibenzothiophene | 0.061 |
|  | C1-dibenzothiophenes | 0.192 |
|  | C2-dibenzothiophenes | 0.265 |
|  | C3-dibenzothiophenes | 0.262 |
|  | C4-dibenzothiophenes | 0.166 |
| 4-6 ring PAHs | Fluoranthene | 0.019 |
|  | Pyrene | 0.086 |
|  | C1-fluoranthrenes/pyrenes | 0.323 |
|  | C2-fluoranthenes/pyrenes | 0.099 |
|  | C3-fluoranthenes/pyrenes | 0.500 |
|  | Benz(a)anthracene | 0.069 |
|  | Chrysene | 0.098 |
|  | C1-chrysenes | 0.482 |
|  | C2-chrysenes | 0.563 |
|  | C3-chrysenes | 0.429 |
|  | C4-chrysenes | 0.275 |
|  | Benzo(b)fluoranthene | 0.021 |
|  | Benzo(k)fluoranthene | 0.003 |
|  | Benzo(e)pyrene | 0.028 |
|  | Benzo(a)pyrene | 0.029 |
|  | Perylene | 0.017 |
|  | Indeno(1,2,3-c,d)pyrene | 0.004 |
|  | Dibenz(a,h)anthracene | 0.008 |
|  | Benzo(g,h,i)perylene | 0.014 |
| C0-C5 phenols | Phenol | 0.004 |
|  | C1-Phenols (o- and p-cresol) | 0.019 |
|  | C2-Phenols | 0.038 |
|  | C3-Phenols | 0.096 |
|  | C4-Phenols | 0.049 |
|  | C5-Phenols | 0.191 |
| Terpanes | 30 ab hopane | 0.068 |
|  |  |  |
| VOC (including BTEX and C3-benzenes) | Isopentane | nd* |
|  | n-C5 (Pentane) | nd |
|  | Cyclopentane | nd |
|  | 2-methylpentane | nd |
|  | 3-Methylpentane | nd |
|  | n-C6 (Hexane) | nd |
|  | Methylcyclopentane | 0.002 |
|  | Cyclohexane | 0.004 |
|  | 2,3-Dimethylpentane | nd |
|  | 3-methylhexane | 0.002 |
|  | n-C7 (Heptane) | 0.004 |
|  | Methylcyclohexane | 0.017 |
|  | 2,4 diethylhexane | Nd |
|  | 2-Methylheptane | 0.005 |
|  | n-C8 (Octane) | 0.028 |
|  | n-C9 (Nonane) | 0.214 |
|  | n-C10 (Decane) | 1.348 |
|  | 1,2,3-Trimethylbenzene | 0.169 |
|  | n-Butylbenzene | 0.087 |
|  | n-Pentylbenzene | 0.036 |
|  | C4-Benzenes | 0.976 |
|  | C5-Benzenes | 0.868 |
| BTEX | Benzene | 0.003 |
|  | Toluene | 0.039 |
|  | Ethylbenzene | 0.025 |
|  | m-Xylene | 0.080 |
|  | p-Xylene | 0.035 |
|  | o-Xylene | 0.048 |
| C3-benzenes | Propylbenzene | 0.047 |
|  | 1-Methyl-3-ethylbenzene | 0.126 |
|  | 1-Methyl-4-ethylbenzene | 0.062 |
|  | 1,3,5-Trimethylbenzene | 0.093 |
|  | 1-Methyl-2-ethylbenzene | 0.070 |
|  | 1,2,4-Trimethylbenzene | 0.374 |
|  | 1,2,3-Trimethylbenzene | 0.169 |
| TEM (µg/mg) |  | 488 |

*nd: not detected (<0.01 ppb)

## 5. References

Faksness, L.-G., Altin, D., Nordtug, T., Daling, P.S., Hansen, B.H., 2015. Chemical comparison and acute toxicity of water accommodated fraction (WAF) of source and field collected Macondo oils from the Deepwater Horizon spill. Marine Pollution Bulletin 91, 222–229. https://doi.org/10.1016/j.marpolbul.2014.12.002

US EPA, 2007. Method 8270 D - Semivolatile organic compounds by gas chromatography/mass spectrometry.

US EPA, 2006. Method 8260 C - Volatile organic compounds by gas chromatography/mass spectrometry (GC/MS).

US EPA, 1986. Method 8100 - Polynuclear Aromatic Hydrocarbons.
